# Supplementary material for: The treatment gap and the HIV care continuum for cisgender men who have sex with men in three South African cities: Findings from a biobehavioural survey, 2019
Source: PLOS Glob Public Health. 2025 Sep 2;5(9):e0004874. doi: 10.1371/journal.pgph.0004874 (PMC12404541; doi:10.1371/journal.pgph.0004874)
Supplement: S1 Appendix — (PDF) [file pgph.0004874.s001.pdf]

Supplementary Appendix 1

ELIGIBILITY SCREENING FORM 1

SURVEY INSTRUMENT 3

Eligibility screening form

INSTRUCTIONS: Complete the entire screening questionnaire for every eligible candidate that comes to survey site for visit #1.

Hello. My name is ____. I would like to first thank you for taking the time to participate in the survey. The person who asked you to participate in the survey may have told you that this survey is about beliefs and perceptions around HIV and behaviors of men. However, before we start the survey, I need to first find out if you are eligible to participate. If you are eligible to participate, then I will introduce you to one of our interviewers who will do a survey with you. Let me also tell you that everything you tell us will be completely confidential. We will not take your name and no one will be able to link your responses to you personally. Do you mind if I start?

(Date)	□□/□□□/□□□□
(Is this person a seed?)	1. YES ____ (Enter Seed Prefix)<br>2. NO
(Coupon # person came in with)	□□□□□<br>(if seed, write 'seed prefix' and '0000')
(Skip if seed) (Does the candidate have a valid coupon?)	Yes<br>No → Ineligible
(Skip if seed) Have you participated in this survey before?	1. YES → Ineligible<br>2. NO
How old are you now?	Age in completed years: _____<br>Under 18 years → ineligible
Now, may I ask about your biological sex? Were you born biologically male or female?	1. Male<br>2. Female → Ineligible
1. How long have you lived, worked, and socialized in (INSERT SURVEY AREA)?	Enter #YEARS ____<br>and #MONTHS ____<br><br>**ELIGIBLE if greater or equal to 6 months**
2. Can you tell me how did you get the coupon you brought here today?	1. Someone I know gave it to me<br>2. A stranger gave it to me<br>3.Found it → **Ineligible**<br>4. Bought or exchanged it for something → **Ineligible**
3. **Interviewer:** (Is participant able to provide verbal informed consent?) (i.e.: not under the influence of alcohol/drugs; able to communicate in mutually understood language).	1. Yes<br>2. No → **Ineligible**
4. Have you ever had oral or anal sex with a man? (If necessary, probe with additional questions to verify knowledge of MSM sexual behavior, see section B below)	1. Yes<br>2. No → **Ineligible**
5. Have you had oral or anal sex with a man in the last **6** months? (If necessary, probe with additional questions to verify knowledge of MSM sexual behavior, see section B below)	1. Yes<br>2. No → **Ineligible**

6. (If there is concern about the individual's eligibility, specifically whether there are questions about whether he is a MSM): **Please ask additional screening questions in section B.**	
7. How confident are you that the participant is truly a member of the target population? (please circle your response)	1. Highly Confident<br>2. Somewhat confident (state reason<br>________________________________<br>3. Not confident<br>________________________________<br>**(please see Supervisor for further instructions)**
**Additional Screening Questions. Check the list below to select which questions to use today**:<br>a. Can you tell me, when someone is on top during oral sex, what is he doing?<br>b. Can you tell me, when someone is on bottom during oral sex, what is he doing?<br>c. Can you tell me, when someone is on top during anal sex, what is he doing?<br>d. Can you tell me, when someone is on bottom during anal sex, what is he doing?<br>e. What do you call it when someone performs both active and passive roles during sex with a man?<br>f.	
Monday: abe Tuesday: cde Wednesday: ace Thursday: bde Friday: abd Saturday: bcd Sunday: bce	
8. (SCREENER: Is the Candidate eligible? (Circle))	1. YES<br>2. NO

**Not eligible** because candidate is: **Mark with 'X' ALL that apply, then END.)**		
	a)	Did not have a valid coupon
	b)	Is under the age of 18
	c)	Participated in the survey before
	d)	Is not a biological male
	e)	Did not have oral or anal sex with a man in the last 6 months
	f)	Has not lived, socialized, or worked in survey area at least 6 months
	g)	Is too drunk/high to do questionnaire
	h)	Unable to communicate in understood language
	i)	Other: ___________________________

Survey Instrument

#	Question	Response Set	Instructions to Questionnaire programmer/interviewer	Comments
**1**	**COUPON INFORMATION AND VALIDATION**			
101.	Enter the candidate's coupon code?	(___|___|___|___|___|		
102.	Enter the candidate's coupon code AGAIN?	(___|___|___|___|___|		
103.	Interviewer ID	(___|___)		
104.	Date of Interview	dd/mmm/yyyy		
105.	Interview Start Time	(___|___:___|___)		
106.	What survey city is this?	1. Johannesburg<br>2. Cape Town<br>3. Mahikeng		
107.	Now, may I ask about your biological sex? Were you born biologically male or female?	1. Yes<br>2. No	If response == No. Verify the response. Participant is not eligible – temporarily stop interview and consult with Site Coordinator	This is a validation question
108.	What is your date of birth? PROBE insert a calculated age then say, "this makes you ___ years old, is this correct?"	(___)(___) / (___)(___)(___)(___)<br>month /year)	If date of birth translated to completed age <18 years on date of interview. Verify the response. Participant is not eligible – temporarily stop interview and consult with Site Coordinator	This is a validation question
109.	Have you had either anal or oral sex with a man in the last 6 months?	1. Yes<br>2. No<br>99. Decline to answer	If response == No or Decline to Answer. Verify the response. Participant is not eligible – temporarily stop interview and consult with Site Coordinator	This is a validation question

#	Question	Response Set	Instructions to Questionnaire programmer/interviewer	Comments
**2**	**DEMOGRAPHICS**			
201.	In what country were you born?	1. Zimbabwe<br>2. Mozambique<br>3.South Africa<br>4. Nigeria<br>5. Botswana<br>6. Namibia<br>7. Lesotho<br>8. Other (specify):<br>88. Don't know<br>99. Decline to answer		
202.	To which race do you belong?	1. Black African<br>2. White<br>3. Coloured<br>4. Indian<br>9. Other (specify): (includes Mixed race, Asian etc.)<br>88. Don't know<br>99. Decline to answer		
203.	What is the highest level of education you completed?	1. Never attended school<br>2. Attended school, but never completed any level<br>3. Primary school (grades 1 - 8)<br>4. Secondary School level<br>5. Tertiary Level<br>6. Other (specify):<br>88. Don’t know<br>99. Decline to answer		
204.	Are you currently enrolled as a student at a tertiary level institution (e.g. university, technical college etc?)	1.Yes<br>2. No<br>99. Decline to answer		

#	Question	Response Set	Instructions to Questionnaire programmer/interviewer	Comments
205.	What is the main source of your income ?<br>DO NOT READ ANSWERS, RECORD ONLY ONE	1. Full-time employment<br>2. Part-time employment<br>3. Self-employed<br>5. Pension<br>6. Social grant<br>7. No source of income<br>8. Other (specify):<br>88. Don't know<br>99. Decline to answer	If No Source of Income – SKIP to 208	
206.	How much money did you personally earn last month? (Total income from ALL sources)	1. Below R1,499<br>2. R1,500- 3,499<br>3. R3,500-9,999<br>4. R10,000 plus<br>88. Don't Know<br>99. Decline to answer		
207.	Including yourself, how many people depend on this income?	(____|____|)		
208.	Is (Johannesburg/Mahikeng/Cape Town – select appropriate survey city) your primary residence?	1. Yes<br>2. No<br>88. Don't know<br>99. Decline to answer	If No - SKIP TO 210	
209.	How long have you lived in (Johannesburg/Mahikeng/Cape Town – select appropriate survey city)?	(____|____) (number of years) (____|____) (and number of months)<br>88. Don't know<br>99. Decline to answer		
209a	In which Zone do you reside in?	1. Zone 1<br>2. Zone2<br>3. Zone 3<br>4. Zone 4		

#	Question	Response Set	Instructions to Questionnaire programmer/interviewer	Comments
209b	In which Zone do you mostly socialise in?			
210.	If your primary residence is not in (Johannesburg/Mahikeng/Cape Town – select appropriate survey city) , where is your primary residence? WRITE-IN response	Free Text ___________________<br>99. Decline to answer		
210a	Do you work in this area?	1. Yes<br>2. No	If No skip to 210d	
210b	How long have worked in this area	(____|____) (number of years) (____|____) (and number of months)<br>88. Don't know<br>99. Decline to answer		
210c	In which Zone do you mostly socialise in?	1. Zone 1<br>2. Zone2<br>3. Zone 3<br>4. Zone 4		
210d	Do you socialize in this area?	1. Yes<br>2. No	If no stop, please confirm eligibility	
210e	How long have worked in this area	(____|____) (number of years) (____|____) (and number of months)<br>88. Don't know<br>99. Decline to answer		
210f	In which Zone do you mostly socialise in?	1. Zone 1<br>2. Zone2<br>3. Zone 3<br>4. Zone 4		
**2A**	**SOCIAL NETWORKS**			

#	Question	Response Set	Instructions to Questionnaire programmer/interviewer	Comments
211	Think about the MSM who you know by sight and/or name and who also know you by sight and/or name.<br>How many are 18 years and above and live/work/socialise in (insert city)?	______________________		
212	*Among the people we know and those who know us, we may have different levels of interaction with each of them. For example: how much time we spend with them, how often we talk to them, or socialise with them. The ones we interact with most are what we call our personal network.*<br><br>Now, thinking of the (response in 211) who you know and also know you, how many are in your personal network?	_____________________		
**3**	**MARRIAGE AND FAMILY**			
301	Have you ever been married or committed to a **woman** as married	1. Yes<br>2. No<br>88. Don't know<br>99. Decline to answer	If No – Go to 303	
301a.	For how long have you been or were you (if not any more)in such a committed relationship?	(____|____) (number of years) (____|____) (and number of months)<br>88. Don't know<br>99. Decline to answer		
302	Are you currently married or committed to a **woman** as married?	1. Yes<br>2. No<br>88. Don't know<br>99. Decline to answer	If No – Go to 303	
302b.	For how long have you been in such a committed relationship?	(____|____) (number of years) (____|____) (and number of months)<br>88. Don't know<br>99. Decline to answer		
303	Have you ever been married or committed to a **man** as married	1. Yes<br>2. No<br>88. Don't know	If No – Go to 401	

#	Question	Response Set	Instructions to Questionnaire programmer/interviewer	Comments
		99. Decline to answer		
303a.	For how long have you been or were you (if not any more) in such a committed relationship?	(____|____) (number of years) (____|____) (and number of months)<br>88. Don't know<br>99. Decline to answer		
304	Are you <u>currently</u> married or committed to a **man** as married	1. Yes<br>2. No<br>88. Don't know<br>99. Decline to answer	If No – Go to 401	
304a.	For how long have you been in such a committed relationship?	(____|____) (number of years) (____|____) (and number of months)<br>88. Don't know<br>99. Decline to answer		
**4**	**SEXUAL HISTORY**	**INTERVIEWER: These next questions are about sexual experiences you may have had. While some people have had a lot of sexual experience, others have not, so questions may or may not apply to you.**		
401	Have you ever had vaginal sex with a woman?	1. Yes<br>2. No<br>99. Decline to answer	SKIP TO Q403	
402	At what age did you first have vaginal sex with a woman?	(____|____) (Age in years)<br>88. Don't Know<br>99. Decline to answer		
403	Have you ever had anal sex with a woman?	1. Yes<br>2. No<br>99. Decline to answer	SKIP TO Q404 (If Q401 & Q403=2 SKIP TO Q408)	
404	In total, with how many different women have you had sex with in the last 6 months? Sex being defined as vaginal or anal sex.<br>If you don't remember, give your best estimate.	(____|____|____) (number of female partners)<br>88. Don't Know<br>99. Decline to answer	IF 0 SKIP TO Q408	

#	Question	Response Set	Instructions to Questionnaire programmer/interviewer	Comments
405	Of these (RESPONSE TO Q404), how many did you NOT use a condom the last time you had sex with them (vaginal or anal sex)?	(____|____|____|____) (number of female partners)<br>88. Don't Know<br>99. Decline to answer		
406	Of these (RESPONSE TO Q405), how many did you give money, goods or services in exchange for sex?	(____|____|____) (number of female partners)<br>88. Don't Know<br>99. Decline to answer		
407	Of these (RESPONSE TO Q405), how many did you receive money, goods or services from in exchange for sex?	(____|____|____) (number of female partners)<br>88. Don't Know<br>99. Decline to answer		
408	Have you ever had anal sex with a man?	1. Yes<br>2. No<br>99. Decline to answer	If no skip to 410	
409	At what age did you first have anal sex with a man?	(____|____) (age in years)<br>88. Don't Know<br>99. Decline to answer		

#	Question	Response Set	Instructions to Questionnaire programmer/interviewer	Comments
410	Where or how do you normally meet male sexual partners?<br>DO NOT READ ANSWERS, RECORD ONLY ONE	1. Intermediary (e.g., friend)<br>2. Bar/Nightclub/Shebeen<br>3. Hotel/Bed & Breakfast<br>4. Street, park or public transport<br>5. Sex-specific mobile dating application/internet (i.e. Grindr, Scruff, manhunt, gaydar)<br>7. Non-sex-specific internet, social media (e.g. Facebook)<br>8. Pride, gay event<br>9. School/university/college<br>10. Not actively searching for male sexual partners<br>11. Other (specify):<br>88. Don't know<br>99. Decline to answer		
**5**	**PARTNER MATRIX**			
501.	In the past 6 months (since MONTH/YEAR), how many people have you had vaginal, oral or anal sex with? Please include males and females, main or casual partners, as well as anyone you gave or received money, goods, or services in exchange for sex.	(____|____|____|____) (Number of partners)<br>88. Don't Know<br>99. Decline to answer	If response = 0; verify response. Participant should have met eligibility criteria for oral or anal sex with man in the last 6 months.	
	INTERVIEWER: Now I am going to ask you a series of questions about the last person with which you had sex (oral, anal or vaginal) (Matrix goes up to last 3 partners based on response to Q501)			
502.	Was this partner biologically male or female?	1. Male<br>2. Female<br>3. Other: specify<br>88. Don't know<br>99. Decline to answer	If Male: SKIP TO Q512	

#	Question	Response Set	Instructions to Questionnaire programmer/interviewer	Comments
503.	How old was your last partner? If you don't know, take your best guess.	(____|____) (age in years)<br>88. Don't Know<br>99. Decline to answer		
504.	Did you give this partner money, goods or services in exchange for sex?	1. Yes<br>2. No<br>88. Don't Know<br>99. Decline to answer		
505.	Did you receive money, goods, or services in exchange for sex?	1. Yes<br>2. No<br>88. Don't Know<br>99. Decline to answer		
506.	What type of partner was your last partner: regular, casual or transactional?<br>(Note to interviewer: if participant has responded Yes to 504 or 505 – then the partner was transactional)	1. Regular partner (committed to the person& no payment or exchange)<br>2. Casual partner (not committed to the person & no payment or exchange)<br>3. Transactional partner (exchanged sex for money, goods, or services)<br>4. Other (specify):<br>88. Don't Know<br>99. Decline to answer		

#	Question	Response Set	Instructions to Questionnaire programmer/interviewer	Comments
507.	Where or how did you meet this partner?<br>DO NOT READ ANSWERS, RECORD ONLY ONE	1. Intermediary (e.g., friend)<br>2. Bar/Nightclub/Shebeen<br>3. Hotel/Bed & Breakfast<br>4. Street, park or public transport<br>5. Sex-specific mobile dating application/internet (i.e. Grindr, Scruff, manhunt, gaydar)<br>7. Non-sex-specific internet, social media (e.g. Facebook)<br>8. Pride, gay event<br>9. School/university/college<br>10. Other (specify):<br>88. Don't know<br>99. Decline to answer		
508.	The last time you had sex with this partner, what did you know or believe this persons HIV status to be?	1. HIV Negative<br>2. HIV Positive<br>88. Don't know<br>99. Decline to answer		
509.	IF FEMALE PARTNER: The last time you had sex (vaginal or anal) with this person was a condom used?	1. Yes<br>2. No<br>3. Not applicable – no vaginal or anal sex<br>99. Decline to answer	If Yes: SKIP TO Q511<br><br>SKIP TO Q518	

#	Question	Response Set	Instructions to Questionnaire programmer/interviewer	Comments
510.	IF FEMALE PARTNER: Could you tell me why a condom was NOT used?<br>DO NOT READ ANSWERS, RECORD ALL MENTIONED	1. No condom available<br>2. Partner refused<br>3. Condom reduces sexual pleasure<br>4. Used other contraceptives<br>5. I am not worried about getting HIV/STIs<br>6. Condoms break / don't work<br>7. Want partner to get pregnant<br>8. I am using PrEP<br>9. I or partner on ART and not worried about transmission<br>10. I or partner have the same HIV status<br>11. Other (specify):<br>88. Don't know<br>99. Decline to answer	SKIP TO SECTION 6 (OR NEXT PARTNER)	
511.	IF FEMALE PARTNER: Can you tell me why a condom was used?<br>DO NOT READ ANSWERS, RECORD ALL MENTIONED	1. To prevent HIV/STIs<br>2. Do not trust partner<br>3. Messages advising the use of condoms<br>4. To prevent pregnancy<br>5. Other (specify):<br>88. Don't know<br>99. Decline to answer		
512.	IF MALE PARTNER: To the best of your knowledge, does this partner also have sex with women?	1. Yes, I know he does<br>2.Yes, he probably does<br>3. No, I know he does not<br>4. No, he probably doesn't<br>88. Don't know<br>99. Decline to answer		
513.	IF MALE PARTNER: To the best of your knowledge, was this partner circumcised?	1. Yes<br>2. No<br>88. Don't know<br>99. Decline to answer		

#	Question	Response Set	Instructions to Questionnaire programmer/interviewer	Comments
514.	IF MALE PARTNER: The last time you had anal sex with this person was it receptive, insertive or both?	1. Insertive<br>2. Receptive<br>3. Both insertive and receptive<br>4. Not applicable (no anal sex with partner)<br>99. Decline to answer	 <br><br>SKIP to Q518	
515.	IF MALE PARTNER: The last time you had anal sex (INSERTIVE OR RECEPTIVE OR BOTH) with this person, was a condom used?	1. Yes<br>2. No<br>88. Don't Know<br>99. Decline to answer	SKIP to Q517	
516.	IF MALE PARTNER: Could you tell me why a condom was NOT used the last time you had anal sex?<br>DO NOT READ ANSWERS, RECORD ALL MENTIONED	1. No condom available<br>2. Partner refused<br>3. Condom reduces sexual pleasure<br>4. I got paid more to not use a condom<br>5. I am not worried about getting HIV/STIs<br>6. Condoms break / don't work<br>7. I am using PrEP<br>8. I or partner on ART and not worried about transmission<br>9. I or partner have the same HIV status<br>10. Other (specify):<br>88. Don’t know<br>99. Decline to answer	SKIP TO SECTION 6 (OR NEXT PARTNER)	
517.	IF MALE PARTNER: Could you tell me why a condom was used the last time you had anal sex?<br>DO NOT READ ANSWERS, RECORD ALL MENTIONED	1. To prevent HIV<br>2. To prevent STIs (other than HIV)<br>2. Do not trust partner<br>3. Messages advising the use of condoms<br>4. Other (specify):<br>88. Don’t know<br>99. Decline to answer		

#	Question	Response Set	Instructions to Questionnaire programmer/interviewer	Comments
518.	FOR THOSE WHO REPORTED NO VAGINAL OR ANAL SEX WITH THIS PARTNER: Did you ONLY have oral sex with this partner in the past 6 months?	1. Yes<br>2. No<br>88. Don't know<br>99. Decline to answer	SKIP TO SECTION 6 (OR NEXT PARTNER)<br>VERIFY RESPONSES	
	**SKIP CHECK: Preceding questions 502-517 to be asked for up to 2additional partners (Refer to Q501 response)**			
**6**	**CONDOM ACCESS AND USE**	**INTERVIEWER: Now I am going to ask you some questions about condom access and your usage of condoms.**		
601.	Could you ask your main/regular sex partner to use a condom if you wanted? (A main/regular sex partner is someone you are committed to, for example your spouse, live-in sex partner, or boyfriend or girlfriend. There is no payment or exchange of goods or services for sex with these partners)	1. Yes<br>2. No<br>88. Don't know<br>99. Decline to answer		
602.	Do you find it very easy, somewhat easy, or not easy to obtain male condoms?	1. Very easy<br>2. Somewhat easy<br>3. Not easy<br>88. Don't Know<br>99. Decline to answer		

#	Question	Response Set	Instructions to Questionnaire programmer/interviewer	Comments
603.	Where do you usually get male condoms?<br>DO NOT READ ANSWERS, RECORD ALL MENTIONED	1. Government hospital/clinic/health centre<br>2. Mobile clinic or mobile outreach<br>3. HIV Testing Services Event (HTS site)<br>4. Private hospital or clinic<br>5. Shop/Supermarket/Spaza<br>6. Pharmacy<br>7. Peer educator or an NGO<br>8. Friends<br>9. Sex partner<br>10. Shebeen or bar<br>11. Never use condoms<br>12. Other (specify):<br>88. Don't know<br>99. Decline to answer		
**7**	**LUBRICANT ACCESS**	**INTERVIEWER: Now, I am going to ask you some questions about lubricant access and your use of lubricant.**<br><br>**IF PARTICIPANT DOES NOT UNDERSTAND WHAT LUBRICANT OR LUBRICATION IS, EXPLAIN THAT IT IS A SUBSTANCE THAT IS USED TO REDUCE THE FRICTION DURING VAGINAL OR ANAL SEX.**		
701.	Have you ever used lubricant during anal sex?	1. Yes<br>2. No<br>88. Don't Know<br>99. Decline to answer	If No: SKIP TO Q704	

#	Question	Response Set	Instructions to Questionnaire programmer/interviewer	Comments
702.	How often did you use lubricant during anal sex in the past 6 months?<br>READ ANSWERS, RECORD ONLY ONE	1. Always<br>2. Usually<br>3. Sometimes<br>4. Rarely<br>5. Never<br>6. Not applicable (no anal sex in the past 6 months)<br>88. Don’t know<br>99. Decline to answer	 <br><br><br>SKIP TO Q704<br>SKIP TO Q704	
703.	What type of lubricant do you usually use?<br>DO NOT READ ANSWERS, RECORD ALL MENTIONED	1. Glycerin<br>2. Saliva or water<br>3. Epizone E<br>4. KY Jelly<br>5. Acqeous Cream/Body lotion/hand lotion<br>6. Magarine/ Butter<br>7. Cooking Oil/ Sunflower Oil<br>8. Vaseline<br>9. Baby oil<br>10. Other oil<br>11. Water-based<br>12. Silicone-based<br>13. Soap<br>14. Whatever we get from peer educator(s), don’t know what it is<br>15. Other (specify):<br>88. Don’t know<br>99. Decline to answer		

#	Question	Response Set	Instructions to Questionnaire programmer/interviewer	Comments
704.	How easy would you say it is to obtain water-based lubricants?<br>READ ANSWERS, RECORD ONLY ONE	1. Very easy<br>2. Somewhat easy<br>3. Not easy<br>4. Don't know what water-lubricants are<br>88. Don't know how easy it is to obtain them<br>99. Decline to answer	 <br><br>SKIP to 801	
705.	Where do you usually obtain water-based lubricants?<br>DO NOT READ ANSWERS, RECORD ALL MENTIONED	1. Government hospital/clinic/health center<br>2. Mobile clinic or mobile outreach<br>3. HIV Testing Services Event (HTS site)<br>4. Private hospital or clinic<br>5. Shop/Supermarket/Spaza<br>6. Pharmacy<br>7. Peer educator or an NGO<br>8. Friends<br>9. Sex partner<br>10. Shebeen or bar<br>12. Other (specify):<br>88. Don't know<br>99. Decline to answer		
**8**	**HEALTHCARE UTILISATION AND STIs**	**Now I am going to ask you some questions about some of your experiences in seeking healthcare. I will also ask you about Sexually Transmitted Infections, also known as STIs. For these questions, we are asking about STIs other than HIV. Please answer to the best of your ability.**		
801.	Where do you normally go for healthcare?	1. Government hospital/clinic/health centre<br>2. Pharmacy<br>3. Private Clinic/GP<br>4. NGO clinic<br>5. Traditional healer<br>6. Other (specify):<br>88. Don't Know<br>99. Decline to answer		

#	Question	Response Set	Instructions to Questionnaire programmer/interviewer	Comments
802.	In the last 6 months have you visited a healthcare facility to receive any medical services?	1. Yes<br>2. No<br>88. Don't Know<br>99. Decline to answer	 If No – skip to Q901	
803.	In the past 6 months, which reasons did you have for visiting a medical care facility ? (Tick all that apply)	1. HIV treatment<br>2. HIV testing<br>3. Receiving treatment for chronic condition (other than HIV)<br>4. Treatment for an injury<br>5. Treatment for an STI<br>6. Mental health<br>7. Other: Specify<br>88. Don't know<br>99. Decline to answer		
804.	In the past 6 months, have you told any health care providers that you have sex with men?	1. No, they didn't ask<br>2. No, they asked, but I didn't tell them<br>3. Yes, they asked<br>4. Yes, they didn't ask, but I told them<br>88. Don't Know<br>99. Decline to answer	 <br>SKIP TO Q806<br>SKIP TO Q806	

#	Question	Response Set	Instructions to Questionnaire programmer/interviewer	Comments
805.	Why didn’t you tell them? (Tick all that apply)	1. I thought the health care provider would make fun of me or treat me differently<br>2. I thought the health care provider would refuse to provide appropriate care for me<br>3. I was uncomfortable talking about having sex with men with my health care provider<br>4. I was uncomfortable talking about sex at all with my health care provider<br>5. I thought friends, family or other people in the community would find out<br>6. I thought it was not important to tell my health care provider<br>88. Don’t Know<br>99. Decline to answer		

#	Question	Response Set	Instructions to Questionnaire programmer/interviewer	Comments
806.	After confirming or revealing that you have sex with other men, did the following happen?<br>(Tick all that apply)	1. The health care provider offered counselling on safer practices while having sex with men<br>2. The health care provider avoided you<br>3. The health care provider made fun of you or treated you differently<br>4. The health care provider refused to help you<br>5. The health care provider offered counselling on how to prevent HIV<br>6. The health care provider asked you about anal itching, anal sores<br>7. The health care provider avoided performing certain screening tests (such as chlamydia or HIV<br>screening) because they felt uncomfortable<br>8. The health care provider offered testing for HIV and STIs<br>9. The health care provider suggested I get care at another place with better services for gay or other men<br>who have sex with men<br>88. Don't Know<br>99. Decline to Answer		
807.	In the past 6 months, have you had difficulty getting medical care when you wanted to get it?	1. Yes<br>2. No<br>88. Don't Know<br>99. Decline to answer	 SKIP TO Q809	

#	Question	Response Set	Instructions to Questionnaire programmer/interviewer	Comments
808.	What difficulty did you have?<br>DO NOT READ ANSWERS, RECORD ALL MENTIONED	1. Too expensive<br>2. Too far away<br>3. Could not take time from work<br>4. Long waiting times<br>5. Stigma/discrimination from healthcare workers<br>5. Other (specify):<br>88. Don't Know<br>99. Decline to answer		
809.	In the last 6 months has a doctor or a medical professional told you that you had a sexually transmitted infection?	1. Yes<br>2. No<br>88. Don't Know<br>99. Decline to answer	 SKIP TO 901	
810.	Sometimes men experience an abnormal discharge from their genitalia AND/OR and ulcer on or near their genitalia or anus.<br><br>In the last 6 months, have you had an abnormal discharge from your genitalia and/or have you had a sore or ulcer on or near your genitalia or anus?	1. Yes<br>2. No<br>88. Don't Know<br>99. Decline to answer		
811.	Did you continue having sexual intercourse during the period when you had an abnormal discharge from your genitalia and/or had a sore or ulcer on or near your genitalia or anus?	1. Yes<br>2. No<br>88. Don't Know<br>99. Decline to answer		
812.	Did you seek treatment for this discharge or ulcer?	1. Yes<br>2. No<br>88. Don't Know<br>99. Decline to answer	SKIP TO Q813	

#	Question	Response Set	Instructions to Questionnaire programmer/interviewer	Comments
813.	Why did you NOT seek treatment?<br>DO NOT READ ANSWERS, RECORD ALL MENTIONED	1. Didn't know where to go for treatment<br>2. Embarrassed or afraid to seek treatment<br>3. Could not afford treatment<br>4. Unable to get transportation<br>5. Didn't think I needed it<br>6. No time to go for treatment<br>7. Other (specify):<br>88. Don't Know<br>99. Decline to answer		
814.	Where did you seek treatment the last time you had discharge, a sore or ulcer?<br>DO NOT READ ANSWERS, RECORD ALL MENTIONED	1. Government clinic<br>2. Private clinic or GP<br>3. NGO run clinic (e.g., Ten81, Health4Men)<br>3. Pharmacy or chemist<br>4. Traditional healer/herbalist<br>5. Medicine or herbs from home<br>6. Other (specify):<br>88. Don't Know<br>99. Decline to answer		
815.	Why did you choose to get treatment from this source/these sources?<br>DO NOT READ ANSWERS, RECORD ALL MENTIONED	1. Confidentiality<br>2. Affordability<br>3. Recommended by friend or acquaintance<br>4. Quality and/or specialized care given at this place<br>5. Knows the caregivers<br>6. Known friendliness of the caregivers<br>7. Proximity/location<br>8. Other (specify):<br>88. Don't know<br>99. Decline to answer		
**9**	**HIV KNOWLEDGE, HIV PREVENTION AND TESTING AND COUNSELLING**		**Now, I am going to ask you some questions about HIV.**	

#	Question	Response Set	Instructions to Questionnaire programmer/interviewer	Comments
901.	Can the chances of getting HIV be reduced by having sex with only one partner who has no other sex partners?	1. Yes<br>2. No<br>88. Don't know<br>99. Decline to answer		
902.	Can a person reduce their chance of getting HIV by using a condom every time they have sex?	1. Yes<br>2. No<br>88. Don't know<br>99. Decline to answer		
903.	Can a person reduce their chance of getting HIV by not having sexual intercourse at all?	1. Yes<br>2. No<br>88. Don't know<br>99. Decline to answer		
904.	Is it possible for a healthy-looking person to have HIV?	1. Yes<br>2. No<br>88. Don't know<br>99. Decline to answer		
905.	Have you heard about special drugs that people infected with HIV can get from a doctor or nurse to help them live longer?	1. Yes<br>2. No<br>88. Don't know<br>99. Decline to answer		
906.	Have you heard about special drugs that people who do not have HIV can get from a doctor or nurse to help them reduce the chances of getting HIV?	1. Yes<br>2. No<br>88. Don't know<br>99. Decline to answer		
**10**	**HIV TESTING HISTORY**	**INTERVIEWER: Now, I am going to ask you questions about HIV testing and your experience. Remember that you do not have to answer any questions you do not feel comfortable answering.**		
1001.	Have you EVER been tested for HIV by a service provider (this can be a health worker, at an event, work, pharmacy etc.)?	1. Yes<br>2. No<br>88. Don't Know<br>99. Decline to answer	SKIP TO Q1003	

#	Question	Response Set	Instructions to Questionnaire programmer/interviewer	Comments
1002.	IF NOT TESTED: Why have you not had an HIV test?<br>DO NOT READ ANSWERS, RECORD ALL MENTIONED.	1. Don’t know where to go<br>2. I always use condoms<br>3. I feel I am not at risk for HIV<br>4. I didn't have time/too busy<br>5. I trust my partner<br>6. Afraid of finding out that I may be HIV positive<br>7. Afraid of people knowing I have gone for testing or that I tested positive<br>8. Inconvenient testing location or hours<br>9. Stigma by healthcare workers<br>10. Lack of confidence in the quality of HIV testing services (e.g. lack of confidentiality, validity of results)<br>9. Other (specify):<br>88 Don’t know<br>99. Decline to answer	SKIP TO Q1012	
1003.	IF TESTED: What was the date of your last HIV test?	(___)(___) / (___)(___)(___)(___) (month /year)<br>888. Don't Know<br>999. Decline to answer		
1004.	IF TESTED: Where was the last test done?	1. Government hospital/clinic/health center<br>2. Private facility/clinic<br>3. Mobile counseling and testing outreach<br>4. School or university<br>5. Work/employer<br>6. Pharmacy (e.g. Clicks, Dischem, etc.)<br>7. An NGO (specify):<br>8. Other (specify):<br>88. Don’t know<br>99. Decline to answer		

#	Question	Response Set	Instructions to Questionnaire programmer/interviewer	Comments
1005.	IF TESTED: For what reasons did you get this last test?<br>DO NOT READ ANSWERS, RECORD ALL MENTIONED	1. Wanted to know my HIV status<br>2. My partner asked me to get tested<br>3. Wanted to start sexual relations with a new partner<br>4. Wanted to get married<br>5. Needed loan/insurance<br>6. Employer requested the test<br>7. I felt sick<br>8. Advised by a health worker<br>9. Advised by a peer educator<br>10. Other (specify):<br>88. Don’t know<br>99. Decline to answer		
1006.	IF TESTED: What was the result of your last HIV test?	1. HIV Negative<br>2. HIV Positive<br>3. Indeterminate<br>4. I didn't get the result<br>88. Don't Know<br>99. Decline to answer	SKIP TO Q1012<br>SKIP TO Q1008<br>SKIP TO Q1012<br><br>SKIP TO Q1012<br>SKIP TO Q1012	
1007.	IF TESTED AND DID NOT GET RESULT: why did you not get your result?	1. I didn't have time/too busy<br>2. I am not infected<br>3. I was too scared<br>4. The testing center didn't have my result<br>5. Other (specify):<br>88. Don’t know<br>99. Decline to answer	SKIP TO Q1012	
1008.	IF POSITIVE, when was your first HIV positive test?	(___)(___) / (___)(___)(___)(___) (month /year)<br>88. Don’t know<br>99. Decline to answer		

#	Question	Response Set	Instructions to Questionnaire programmer/interviewer	Comments
1009.	IF POSITIVE, What do you think your chances of transmitting HIV to a partner is? No chance, small, moderate or great chance?	1. No chance<br>2. Small chance<br>3. Moderate chance – SKIP TO Q1010<br>4. Great chance – SKIP TO Q1011<br>88. Don't Know<br>99. Decline to answer		
1010.	Why do you think you have no chance/small chance of transmitting HIV to a partner?<br>DO NOT READ ANSWERS, RECORD ALL MENTIONED	1. Never had anal sex<br>2. Currently abstaining from sex<br>3. Fidelity to Partner/Trust in partner<br>4. Use condoms<br>5. Undetectable/virally suppressed<br>6. I am the "bottom" during anal sex<br>7. I am the "top" during anal sex<br>8. I pull out before ejaculation<br>9. I only have sex with other HIV-positive people<br>10. I do not inject drugs with partners<br>11: Other, specify:<br>88. Don't know<br>99. Decline to answer		
1011.	Why do you think you have a moderate chance/great chance of transmitting HIV to a partner?<br>DO NOT READ ANSWERS, RECORD ALL MENTIONED	1. I don't use condoms<br>2. Multiple partners<br>3. Infidelity/don't trust in partner<br>4. I inject drugs with partners<br>5. High viral load count<br>6. I am the "top" during anal sex<br>7. I am the "bottom" during anal sex<br>8. I don't pull out before ejaculation<br>9. I have sex with HIV-negative people<br>10. I have injuries/cuts<br>11. Other, specify:<br>88. Don't know<br>99. Decline to answer		

#	Question	Response Set	Instructions to Questionnaire programmer/interviewer	Comments
1012.	What do you think are your chances of getting HIV? No chance, small, moderate or great chance?	1. No chance<br>2. Small chance<br>3. Moderate chance<br>4. Great chance<br>88. Don't know<br>99. Decline to answer	SKIP TO Q1013<br>SKIP TO Q1013<br>SKIP TO Q1014<br>SKIP TO Q1014	
1013.	Why do you think that you have no chance/small chance of getting HIV?<br>DO NOT READ ANSWERS, RECORD ALL MENTIONED	1. Never had anal sex<br>2. Currently abstaining from sex<br>3. Fidelity to Partner/Trust in partner<br>4. Use condoms<br>5. Other (specify):<br>88. Don't know<br>99. Decline to answer	SKIP TO Q1015	
1014.	Why do you think that you have a moderate chance/great chance of getting HIV?<br>DO NOT READ ANSWERS, RECORD ALL MENTIONED	1. Don't use condoms<br>2. Don't trust partner<br>3. Had injuries/cuts<br>4. Multiple partners<br>5. I am injecting drugs<br>6. Other (specify):<br>88. Don't know<br>99. Decline to answer		
1015.	What do you think your HIV status is today?	1. HIV Negative<br>2. HIV Positive<br>88. Don't know<br>99. Decline to answer	IF Q1002 = 2 SKIP TO instruction before Q1020	
INTERVIEWER: The next few questions ask about HIV self-tests. An HIV self-test usually refers to a rapid HIV test that you can give yourself at home or any convenient location by collecting your own blood (pricking your finger with a small needle) or saliva. If you test HIV-positive on an HIV self-test, you will still need to take a standard HIV test at a hospital, clinic or other organization to confirm your diagnosis.				
1016.	Have you ever heard of HIV self-testing/screening?	1. Yes<br>2. No<br>88. Don't Know<br>99. Decline to answer	If NO: SKIP TO SECTION 11	

#	Question	Response Set	Instructions to Questionnaire programmer/interviewer	Comments
1017.	Have you ever done an HIV test on your own? That is, collected your own saliva or blood and read your own result?	1. Yes<br>2. No<br>88. Don't Know<br>99. Decline to answer		
1018.	IF SELF-TESTED/SCREENED: What was the date of your last HIV self-test/screen?	(___)(___) / (___)(___)(___)(___) (month /year)<br>888. Don't Know<br>999. Decline to answer		
1019.	IF SELF-TESTED/SCREENED: Where did you get the HIV self-test/screening kit for the most recent test/screen you performed?<br><br>DO NOT READ ANSWERS, RECORD ALL MENTIONED	1.Private Pharmacy<br>2.Government Clinic/Hospital<br>3. NGO/peer outreach workers<br>4. Partner (regular, causal, transactional)<br>5. Friend<br>6. Shop/Tuckshop/Spaza<br>7. Other (specify)<br>88. Don't Know<br>99. Decline to answer		
1020.	IF SELF-TESTED/SCREENED: What was the result of your last HIV self-test/screen?	1.HIV Positive<br>2.HIV Negative<br>3. No HIV result<br>88. Don't Know<br>99. Decline to answer		

#	Question	Response Set	Instructions to Questionnaire programmer/interviewer	Comments
1021.	In South Africa, HIV Positive results from a self-test/screen need to be confirmed using two other HIV test devices approved by the government.<br><br>IF POSITIVE: did you go to a healthcare provider for additional testing to confirm the HIV positive result.	1. Yes<br>2. No<br>88. Don't Know<br>99. Decline to answer		
1022.	If HIV self-test kits were available, how likely is it that you would use one to test yourself for HIV?	1. Very unlikely<br>2. Somewhat unlikely<br>3. Neutral<br>4. Somewhat likely<br>5. Very likely<br>6. Not applicable (already known HIV positive)<br>88. Don't know<br>99. Decline to answer		
**11**	**HIV CARE AND TREATMENT**	**INTERVIEWER: Because you have said you know your HIV status to be positive, I am now going to ask you some questions about HIV treatment.**		
1101.	IF POSITIVE: Have you seen a nurse, doctor or other health care provider for a medical evaluation or care related to your HIV infection?	1. Yes<br>2. No<br>88. Don't Know<br>99. Decline to answer	If YES: SKIP TO Q1103	

#	Question	Response Set	Instructions to Questionnaire programmer/interviewer	Comments
1102.	Why have you never seen a nurse, doctor, or other health care provider for a medical evaluation or care related to your HIV infection?	1. Feel fine / healthy<br>2. Don't know where to go<br>3. Too expensive<br>4. Privacy/confidentiality<br>5. Previous bad experience with healthcare<br>6. Don't want to think about being HIV +<br>7. Afraid of people knowing that I am HIV positive<br>8. Inconvenient clinic location or hours<br>9. Stigma by healthcare workers for being MSM<br>10. Lack of confidence in the quality of HIV treatment services (e.g. negative healthcare provider attitudes)<br>11. Other (specify)<br>88. Don't Know<br>99. Decline to Answer		
1103.	Have you had a viral load test?	1. Yes<br>2. No<br>88. Don't Know<br>99. Decline to answer	IF NO: SKIP TO Q1106	
1104.	When was your last viral load test?	(___)(___) / (___)(___)(___)(___) (month /year)<br>888. Don't know<br>999. Decline to answer		
1105.	What was your last viral load?	1. Less than 401 copies/undetectable<br>2. 401-1000 copies<br>3. 1001-10,000 copies<br>4. 10,001-100,000 copies<br>5. More than 100,000 copies<br>88. Don't Know<br>99. Decline to answer		

#	Question	Response Set	Instructions to Questionnaire programmer/interviewer	Comments
1106.	Have you ever been on antiretroviral (ARVs) therapy or ART (medication to treat your HIV infection?)	1. Yes<br>2. No<br>88. Don't Know<br>99. Decline to answer	IF YES SKIP TO Q1108	
1107.	Why have you never been on ART?	1. Feel fine / healthy<br>2. Don't know where to get them<br>3. I cannot afford them<br>4. Concerned about side effects<br>5. Health provider advised not to take<br>6. Don't want to think about being HIV +<br>7. Other: Specify<br>88. Don't Know<br>99. Decline to answer		
1108.	Are you currently on ART?	1. Yes<br>2. No<br>88. Don't Know<br>99. Decline to answer	IF YES: SKIP TO Q1111	
1109.	Why did you stop ART?<br>READ ANSWERS, RECORD ALL MENTIONED	1. They made me sick<br>2. They did not work<br>3. I could not afford them<br>4. Distance to get them is far/queues too long<br>5. I was feeling better and did not need them<br>6. A doctor/nurse told me to stop taking them<br>7. The pharmacy/clinic ran out of the medicine<br>8. I missed my appointment/I ran out of the medication<br>9. Other (specify):<br>88. Don't Know<br>99. Decline to answer		

#	Question	Response Set	Instructions to Questionnaire programmer/interviewer	Comments
1110.	If no longer on ART, where did you go for ART?<br>READ ANSWERS, RECORD ALL MENTIONED	1. Government hospital/clinic/health centre<br>2. Private pharmacy (CCMMDD – government programme)<br>3. Private Clinic/GP/pharmacy (self-paying)<br>4. Community adherence club<br>5. Other community pick up points (i.e. hall or church)<br>6. NGO clinic (e.g., Men4Health or Ten81)<br>7. Traditional healer<br>8. Other (specify):<br>88. Don't Know<br>99. Decline to answer		
1111.	If on ART, where do you go for ART?<br>READ ANSWERS, RECORD ALL MENTIONED	1. Government hospital/clinic/health centre<br>2. Pick up of pre-packaged medication from private pharmacy (government programme)<br>4. Pick up of pre-packaged medication from community adherence club<br>5. Pick up of pre-packaged medication from community venue (i.e. hall or church with no group sessions)<br>6. NGO clinic (e.g., Men4Health or Ten81)<br>3. Private Clinic/GP/pharmacy (self-paying)<br>7. Traditional healer<br>8. Other (specify):<br>88. Don't Know<br>99. Decline to answer		
1112.	Thinking about ARVs you take: How many pills are you supposed to take each day?	Enter a whole number: ___ ____		
1113.	Please think back at the last WEEK. Please tell me how many pills have you missed?	Enter a whole number: ___ ____		
1114.	How about in the last MONTH. How many pills have you missed since then? (Interviewer, please make sure the patient is thinking about the last month. You may	Enter a whole number: ___ ____		

#	Question	Response Set	Instructions to Questionnaire programmer/interviewer	Comments
	have to help the participant with the timeframe)			
	(READ to participant): **Thinking about last month, could you show me on this line approximately what proportion of your pills you took in the last month? An x on this end** (point to left end, 0) **means that you didn't take any of your pills. An x on this end** (point to right end, 100) **means that you took every one of your pills and didn't miss a single one. An x here** (point to the middle of the line, 50) **means that you took half of your pills and missed half of your pills.**<br><br>INTERVIEWER: When asking the following question, please show the participant the scale showing a line going from 0 to 100.<br>**0---------|---------|---------|---------|---------|---------|---------|---------|---------|---------|---------100**<br>**10 20 30 40 50 60 70 80 90**			
1115.	Please point to the spot on the line that best represents the proportion of pills you took last month.	Use the scale above		
1116.	Since you first started taking your HIV medication, how many times have you gone for at least 2 days without taking them?	____ (# of times)		
1117.	When was the last time that this happened?	_ _ / _ _ _ _ (mm/yyyy)		
1118.	At that time, how many days did you go without taking your HIV medication?	____ (# of days)		
1119.	What is the longest time you have ever gone without taking your HIV medication?	____ (# of days)		
1120.	How many times have you gone at least 2 days without taking HIV medication in the last 3 months?	____ (# of times)		
**12**	**TUBERCULOSIS**			
1201.	*In South Africa people are screened for TB by asking four questions – whether they have fever for 2 weeks, cough, night sweats or weight loss. People may also provide sputum for lab testing or have chest X rays done to check for TB.*<br><br>Have you ever been screened or tested for TB?	1. Yes<br>2. No	If NO: SKIP TO Q1301	

#	Question	Response Set	Instructions to Questionnaire programmer/interviewer	Comments
		88. Don't Know<br>99. Decline to answer		
1202.	Have you ever been diagnosed with TB disease?	1. Yes<br>2. No<br>88. Don't Know<br>99. Decline to answer	If NO: SKIP TO Q1301	
1203.	When was the last time you were diagnosed with TB disease?	(___)(___) / (___)(___)(___)(___) (month /year)<br>888. Don’t know<br>999. Decline to answer		
1204.	Did you receive medication for last time you were diagnosed with TB disease?	1. Yes<br>2. No<br>88. Don't Know<br>99. Decline to answer	If NO: SKIP TO Q1301	
1205.	Did you finish taking all your medication required treating TB disease the last time you were diagnosed?	1. Yes<br>2. No<br>88. Don't Know<br>99. Decline to answer	If YES: SKIP TO Q1301	

#	Question	Response Set	Instructions to Questionnaire programmer/interviewer	Comments
1206.	Why did you not complete the TB treatment?<br>DO NOT READ ANSWERS. RECORD ALL MENTIONED	1. They made me sick<br>2. They did not work<br>3. I could not afford them<br>4. Distance to get them is far<br>5. Queues at the clinic too long<br>6. I was feeling better and did not need them<br>7. A doctor/nurse told me to stop taking them<br>8. The pharmacy/clinic ran out of the medicine<br>9. I am still taking the medication<br>10. Other (specify):<br>88. Don't Know<br>99. Decline to answer		
**13**	**STIGMA DISCRIMINATION AND VIOLENCE**	**INTERVIEWER: Now I will ask you some questions about discrimination and violence. While some people may have experienced these, others may not. Please remember your answers will be kept private. (To the interviewer: Remember to offer referrals for any responses that suggest violence)**		
	ENACTED MSM STIGMA (NON-HEALTHCARE): In the past 6 months, how often have the following happened to you because you are a man who has sex with other men?			
1301.	Hit or beaten up?	1. Never<br>2. Once<br>3. 2-3 times<br>4. 4 or more times<br>5. Does not apply	If never go to Q1303	
1302.	Who was the person who last hit, kicked, or beat you?	1. Do not know the person<br>2. Social acquaintance<br>3. Family/relative<br>4. Police<br>5. Client<br>6. Sexual partner/lover<br>7. Other (specify):<br>88. Don't Know<br>99. Decline to answer		

#	Question	Response Set	Instructions to Questionnaire programmer/interviewer	Comments
1303.	Treated rudely or unfairly?	1. Never<br>2. Once<br>3. 2-3 times<br>4. 4 or more times<br>5. Does not apply		
1304.	Made fun of or called names?	1. Never<br>2. Once<br>3. 2-3 times<br>4. 4 or more times<br>5. Does not apply		
1305.	Felt uncomfortable in a crowd of other MSM?	1. Never<br>2. Once<br>3. 2-3 times<br>4. 4 or more times<br>5. Does not apply		
1306.	Lost employment or dismissed from a job?	1. Never<br>2. Once<br>3. 2-3 times<br>4. 4 or more times<br>5. Does not apply		
1307.	Rejected by family members?	1. Never<br>2. Once<br>3. 2-3 times<br>4. 4 or more times<br>5. Does not apply		
1308.	Excluded from activities traditionally reserved for men?	1. Never<br>2. Once<br>3. 2-3 times<br>4. 4 or more times<br>5. Does not apply		

#	Question	Response Set	Instructions to Questionnaire programmer/interviewer	Comments
1309.	Someone physically forced you to have sex with them?	1. Never<br>2. Once<br>3. 2-3 times<br>4. 4 or more times<br>5. Does not apply	If never go to Q1311	
1310.	Who was the last person who physically forced you to have sex with them?	1. Do not know the person<br>2. Social acquaintance<br>3. Family/relative<br>4. Police<br>5. Client<br>6. Sexual partner/lover<br>7. Other (specify):<br>88. Don't Know<br>99. Decline to answer		
	MSM INTERNALIZED STIGMA: How do you feel about being a MSM? Please rate how much you agree with the following statements.			
1311.	If I could change being a MSM to be a man who has sex only with women, I would do it.	1. Disagree<br>2. Neutral<br>3. Agree		
1312.	If people call me names, I am good at ignoring it	1. Disagree<br>2. Neutral<br>3. Agree		

#	Question	Response Set	Instructions to Questionnaire programmer/interviewer	Comments
1313.	I feel ashamed of being a MSM	1. Disagree<br>2. Neutral<br>3. Agree		
1314.	Social involvement with other MSM makes me feel uncomfortable	1. Disagree<br>2. Neutral<br>3. Agree		
1315.	I feel I am not as good as others because I am a MSM	1. Disagree<br>2. Neutral<br>3. Agree		
1316.	I think less of myself when I am in public with a person who is obviously a MSM	1. Disagree<br>2. Neutral<br>3. Agree		
1317.	I think being a MSM is against the will of God	1. Disagree<br>2. Neutral<br>3. Agree		
1318.	I perceive myself as physically and emotionally weak because I am a MSM	1. Disagree<br>2. Neutral<br>3. Agree		

#	Question	Response Set	Instructions to Questionnaire programmer/interviewer	Comments
	In the past 6 months, how often have the following happened to you because someone knew or assumed you were a MSM?			
1319.	I avoided going to healthcare services	1. Never<br>2. Once<br>3. 2-3 times<br>4. 4 or more times<br>5. Does not apply		
1320.	I was denied healthcare services	1. Never<br>2. Once<br>3. 2-3 times<br>4. 4 or more times<br>5. Does not apply		
1321.	I was not treated well when receiving healthcare services	1. Never<br>2. Once<br>3. 2-3 times<br>4. 4 or more times<br>5. Does not apply<br>5. Does not apply		
1322.	Someone lured me on a social media platform and I experienced stigma, discrimination or violence	1. Never<br>2. Once<br>3. 2-3 times<br>4. 4 or more times<br>5. Does not apply<br>5. Does not apply		
**14**	**PEP AND PrEP**			

#	Question	Response Set	Instructions to Questionnaire programmer/interviewer	Comments
1401.	*PEP is the use of special medication or drugs used after a single high-risk event to help stop HIV from making copies of itself and spreading through your body. PEP must be taken for 28 days and must be started as soon as possible to be effective—and always within 3 days of a possible exposure.*<br><br>Before today, have you previously heard about taking pills used to treat HIV for 28 days after an exposure in order to prevent HIV infection?	1. Yes<br>2. No<br>88. Don't Know<br>99. Decline to Answer	IF NO: SKIP TO Q1405	
1402.	Have you ever taken PEP?	1. Yes<br>2. No<br>88. Don't Know<br>99. Decline to Answer	IF NO: SKIP TO Q1405	
1403.	In the past 6 months you ever taken PEP?	1. Yes<br>2. No<br>88. Don't Know<br>99. Decline to Answer	IF NO: SKIP TO Q1405	
1404.	Why did you use PEP?<br>(multiple responses allowed – Do not mention responses to participants)	1. I had unprotected sex<br>2. I was raped/forced to have sex<br>3. I shared needles<br>4. Other specify<br>88. Don't Know<br>99. Decline to Answer		

#	Question	Response Set	Instructions to Questionnaire programmer/interviewer	Comments
1405.	***PrEP,*** *is a way for people who do not have HIV but who are at substantial risk of getting it to prevent HIV infection by taking a pill every day. When someone is exposed to HIV through sex or injection drug use, these medicines can work to keep the virus from establishing a permanent infection.*<br><br>Before today, have you heard about taking a pill every day to prevent HIV infection?	1. Yes<br>2. No<br>88. Don't Know<br>99. Decline to Answer	IF NO: SKIP TO Q1501	
1406.	Have you ever taken PrEP; a pill taken every day to prevent HIV infection?	1. Yes<br>2. No<br>88. Don't Know<br>99. Decline to Answer	IF YES: SKIP TO Q1409	
1407.	What is the main reason you have never taken PrEP	1. Embarrassed to talk about it with doctor/nurse<br>2. Don't feel at risk for HIV<br>3. Not available where I live<br>4. Don't know where to get it<br>5. Don't want it<br>6. Afraid of side effects<br>7. Don't want other to know<br>8. Other (specify)<br>88. Don't Know<br>99. Decline to Answer		
1408.	PrEP has similar side effects to other drugs used to treat HIV. Would you Take PrEP to help prevent HIV	1. Yes<br>2. No<br>88. Don't Know<br>99. Decline to Answer		

#	Question	Response Set	Instructions to Questionnaire programmer/interviewer	Comments
1409.	Are you currently using PrEP; a pill every day to prevent HIV infection?	1. Yes<br>2. No<br>88. Don't Know<br>99. Decline to Answer	If Yes: Skip to Q1412	
1410.	In the last 6 months have you taken PrEP	1. Yes<br>2. No<br>88. Don't Know<br>99. Decline to Answer		
1411.	What is the main reason you stopped taking PrEP?	1. I trust my partners<br>2. Can't get PrEP anymore<br>3. Had side effects<br>4. Don't want others to know<br>5. Tested HIV positive<br>6. Other (specify)<br>88. Don't Know<br>99. Decline to Answer		
1412.	From where do you usually get PrEP?	1. Government clinic/hospital<br>2. Private clinic/hospital<br>3. Community based NGO<br>4. Peer educators<br>5. Private Pharmacy<br>6. Friends<br>7. Other (specify)<br>88. Don't Know<br>99. Decline to Answer		
**15**	**CIRCUMCISION**			

#	Question	Response Set	Instructions to Questionnaire programmer/interviewer	Comments
1501.	Have you been circumcised (if circumcised also ask what method and select appropriate response)?<br><br>Interviewer: SHOW THE CARD WITH PICTURE OF CIRCUMCISED AND UNCIRCUMCISED PENIS.	1. Yes, medical circumcision<br>2. Yes, traditional circumcision<br>3. Yes (don’t know whether medical or traditional)<br>2. No<br>99. Decline to answer	If Yes, SKIP to 1316	
1502.	What is the main reason why you have not been circumcised?	1. Never considered it<br>2. Too painful<br>3. Don’t know where to go<br>4. Costs too much<br>5. Religious reasons<br>6. I don’t think I need it<br>7. Other (specify):<br>88. Don’t know<br>99. Decline to answer		
**16**	**PROGRAM COVERAGE**			
1601.	In the last 6 months, did you receive any of these items for free?<br>READ ANSWERS, RECORD ALL MENTIONED	1. Condoms<br>2. Lubricants<br>3. Pamphlets<br>4. PrEP<br>5. HIV Self-test/screen tests<br>6. None<br>7. Other (specify):<br>88. Don't Know<br>99. Decline to answer	IF NONE: SKIP TO Q1606	
1602.	Which organization(s) gave these items?<br>RECORD ALL MENTIONED	1. Health4Men<br>2. AURUM<br>3. ANOVA<br>4. Gay Umbrella<br>5. OUT Wellbeing<br>88. Don't Know<br>99. Decline to answer		

#	Question	Response Set	Instructions to Questionnaire programmer/interviewer	Comments
1603.	In the last 6 months, have you been in contact with a peer educator in the community?	1. Yes<br>2. No<br>88. Don't Know<br>99. Decline to answer	 SKIP TO Q1701	
1604.	Which organization(s) or institution(s) were sponsoring these peer educators?<br>RECORD ALL MENTIONED.	1. Health4Men<br>2. AURUM<br>3. ANOVA<br>4. Gay Umbrella<br>5. OUT Wellbeing<br>88. Don't Know<br>99. Decline to answer		
1605.	How many times have you been in contact with the peer educator in the last 6 months?	(__|__|__) (number of times)<br>88. Don't Know<br>99. Decline to answer		
1606.	What services or information did you receive from the peer educator?<br>DO NOT READ ANSWERS, RECORD ALL MENTIONED	1. Information on HIV/STI prevention/transmission, PrEP,<br>2. Condoms<br>3. Lubricant<br>3. Referral for STI Treatment<br>4. Referral or provision of HIV rapid testing<br>5. Psychosocial support<br>6. Peer navigation/accompany one to clinic<br>7. HIV self-test/self-screening tests<br>8. Other (specify):<br>88. Don't Know<br>99. Decline to answer		
**17**	**ALCOHOL AND DRUG USE**	**INTERVIEWER: Now, I would like to ask some questions about your alcohol and drug use.**		

#	Question	Response Set	Instructions to Questionnaire programmer/interviewer	Comments
1701.	During the past 6 months, how often did you have a drink containing alcohol?<br>(By drink we mean a 350ml can or glass of beer or cider, a standard glass of wine, or a drink containing 1 shot of liquor)<br>DO NOT READ ANSWERS	1. Never<br>2. Monthly or less<br>3. 2-4 times a month<br>4. 2-3 times a week<br>5. 4 or more times a week<br>88. Don't Know<br>99. Decline to answer	IF NEVR: SKIP TO Q1704	
1702.	During the past 6 months, how many drinks containing alcohol did you have on a typical day when you were drinking?<br>DO NOT READ ANSWERS	1. 1 or 2<br>2. 3 or 4<br>3. 5 or 6<br>4. 7 or 9<br>5. 10 or more<br>88. Don't Know<br>99. Decline to answer		
1703.	During the past 6 months, how often did you have six or more drinks on one occasion?<br>DO NOT READ ANSWERS	1. Never<br>2. Less than monthly<br>3. Monthly<br>4. Weekly<br>5. Daily or almost daily<br>88. Don't Know<br>99. Decline to answer		
1704.	*INTERVIEWER: Some people have tried a range of different types of drugs. How often have you used the following drugs in the past 6 months*<br>HAVE RESPONDENT ANSWER FOR EACH SEPARATELY<br>1. Heroin<br>2. TIK<br>3. Ecstasy<br>4. Marijuana<br>5. Cat<br>6. Crystal Meth<br>7. Alcohol<br>8. Cocaine	Frequency Table<br>1. Did not use this drug in the last 6 months<br>2. Monthly or less<br>3. Several times a month<br>4. Two to four times a month<br>5. Two to three times a week<br>6. Four or more times a week<br>7. Never used drug<br>88. Don't Know<br>99. Decline to answer		

#	Question	Response Set	Instructions to Questionnaire programmer/interviewer	Comments
	9. Nyaope<br>10. Mandrax<br>11. Wunga			
1705.	Some people have tried injecting drugs using a syringe or needle. Have you ever injected drugs before today?	1. Yes<br>2. No<br>88. Don't Know<br>99. Decline to answer	IF NO: SKIP TO Q1801	
1706.	Some people have tried injecting drugs using a syringe or needle. Have you injected drugs in the past 6 months?	1. Yes<br>2. No<br>88. Don't Know<br>99. Decline to answer	IF NO: SKIP TO Q1801	
1707.	How frequently did you inject drugs in the past 6 months?	1. Monthly or less<br>2. Two to four times a month<br>3. Two to three times a week<br>4. Four or more times a week<br>88. Don't Know<br>99. Decline to answer		
1708.	What drug do you normally inject? RECORD ALL MENTIONED	1. Heroin<br>6. Crystal Meth/Tik<br>8. Cocaine<br>10. Other (specify):<br>88. Don't Know<br>99. Decline to answer		
1709.	Have you shared a syringe or needle with anyone else when injecting drugs in the last 6 months?	1. Yes<br>2. No<br>88. Don't Know<br>99. Decline to answer		

#	Question	Response Set	Instructions to Questionnaire programmer/interviewer	Comments
1710.	Are you aware of any drug treatment programme that may be available for you?	1. Yes<br>2. No<br>88. Don't Know<br>99. Decline to answer		
1711.	Have you ever been in a drug treatment programme?	1. Yes<br>2. No<br>88. Don't Know<br>99. Decline to answer		
1712.	In the last 6 months, have you ever been in a drug treatment programme?	1. Yes<br>2. No<br>88. Don't Know<br>99. Decline to answer		
1713.	In the last 6 months did you sell sex for drugs?	1. Yes<br>2. No<br>88. Don't Know<br>99. Decline to answer		
**18**	**GENDER/SEX/SEXUALITY**	**The following questions are about how you think about yourself in terms of your sex and your sexuality.**		
1801.	Do you currently feel more sexually attracted to men or to women?	1. Only to women<br>2. More to women than to men<br>3. To women and men equally<br>4. More to men than to women<br>5. Only to men<br>88. Don’t know<br>99. Decline to answer		

#	Question	Response Set	Instructions to Questionnaire programmer/interviewer	Comments
1802.	What word would you use to describe your sexuality?	1. Gay<br>2. Homosexual<br>3. Bisexual<br>4. Straight<br>5. Heterosexual<br>8. Other, please specify:<br>88. Don’t know<br>99. Decline to answer		
1803.	What word would you use to describe your gender?	1. Man<br>2. Woman<br>3. Transgender<br>8. Other, please specify:<br>88. Don’t know<br>99. Decline to answer		
	How do you see yourself in terms of masculinity and femininity? "Masculine" refers to men or women who feel, look and act like “real” men or in a manner which most people think that men should be like. "Feminine" is the opposite of masculine and refers to what usually is expected from women. People, men or women, who look and behave like “real” women are called feminine. Indicate on a scale from "not at all" to "extremely" how masculine and feminine you think you are.			
1901.	In general, how masculine do you think you are?	1. Not at all<br>2. Very little<br>3. Fairly<br>4. Very much<br>5. Extremely		
1902.	In general, how masculine do you act and behave?	1. Not at all<br>2. Very little<br>3. fairly<br>4. Very much<br>5. Extremely		

#	Question	Response Set	Instructions to Questionnaire programmer/interviewer	Comments
1903.	How masculine do you think you appear and come across to others?	1. Not at all<br>2. Very little<br>3. Fairly<br>4. Very much<br>5. Extremely		
1904.	In general, how feminine do you think you are?	1. Not at all<br>2. Very little<br>3. Fairly<br>4. Very much<br>5. Extremely		
1905.	In general, how feminine do you act and behave?	1. Not at all<br>2. Very little<br>3. Fairly<br>4. Very much<br>5. Extremely		
1906.	How feminine do you think you appear and come across to others?	1. Not at all<br>2. Very little<br>3. Fairly<br>4. Very much<br>5. Extremely		
1907.	Have you ever thought of yourself as a woman?	1. Yes<br>2. No<br>88. Don't Know<br>99. Decline to answer		
**20**	**POPULATION SIZE ESTIMATION**			
2001.	Before we started the survey we passed out some objects in the community. This was done by outreach workers and they may have told you to keep it and not give to anyone. Did you receive any of the object shown on this card? (Interviewer, show participant the card with picture of many objects)?	1. Shows the right object<br>2. Shows the wrong object<br>NO<br>3. Cannot show/don’t know<br>99. Decline to answer		

#	Question	Response Set	Instructions to Questionnaire programmer/interviewer	Comments
2002.	On {Date) did you attend an event hosted by (organization) held at (location name).	1. Yes<br>2. No<br>88. Don't Know<br>99. Decline to answer		
Between January 1 and June 15, 2019 did you...				
(Johannesburg (Gauteng) Site)				
2003.	...receive HIV services at Anova – Health4Men?			
(North West (Mahikeng) Site)				
2004.	receive service from Gay Umbrella			
(Cape Town (Western Cape) Site)				
2005.	…receive service at the Ivan Toms Centre for Men's Health?			
