# Supplementary material for: The treatment gap and the HIV care continuum for cisgender men who have sex with men in three South African cities: Findings from a biobehavioural survey, 2019
Source: PLOS Glob Public Health. 2025 Sep 2;5(9):e0004874. doi: 10.1371/journal.pgph.0004874 (PMC12404541; doi:10.1371/journal.pgph.0004874)
Supplement: S2 Appendix — (PDF) [file pgph.0004874.s002.pdf]

Supplementary Appendix 2

Contents

Survey recruitment flow diagrams 1

RDS recruitment trees 3

Recruitment diagnostics 5

Supplementary tables 8

List of tables

Table 1: Cascade Variables by Participant Characteristics - Cape Town (Unweighted) 8
Table 2: Cascade Variables by Participant Characteristics - Cape Town (Weighted) 9
Table 3: Cascade Variables by Participant Characteristics - Johannesburg (Unweighted) 9
Table 4: Cascade Variables by Participant Characteristics - Johannesburg (Weighted) 10
Table 5: Cascade Variables by Participant Characteristics - Mahikeng (Unweighted) 12
Table 6: Cascade Variables by Participant Characteristics - Mahikeng (Weighted) 13

List of figures

Figure 1: Cape Town recruitment flow diagram 1
Figure 2: Johannesburg recruitment flow diagram 1
Figure 3: Mahikeng recruitment flow diagram 2
Figure 4: RDS recruitment graph for Cape Town 3
Figure 5: RDS recruitment graph for Johannesburg 4
Figure 6: RDS recruitment graph for Johannesburg 4
Figure 7: Bottleneck and convergence plots of HIV prevalence in Cape Town 5
Figure 8: Bottleneck and convergence plots of HIV prevalence in Johannesburg 5
Figure 9: Bottleneck and convergence plots of HIV prevalence in Mahikeng 6
Figure 10: Convergence plot of the 95-95-95 care cascade for Cape Town 6
Figure 11: Convergence plot of the 95-95-95 care cascade for Johannesburg 6
Figure 12: Convergence plot of the 95-95-95 care cascade for Johannesburg 7

Survey recruitment flow diagrams

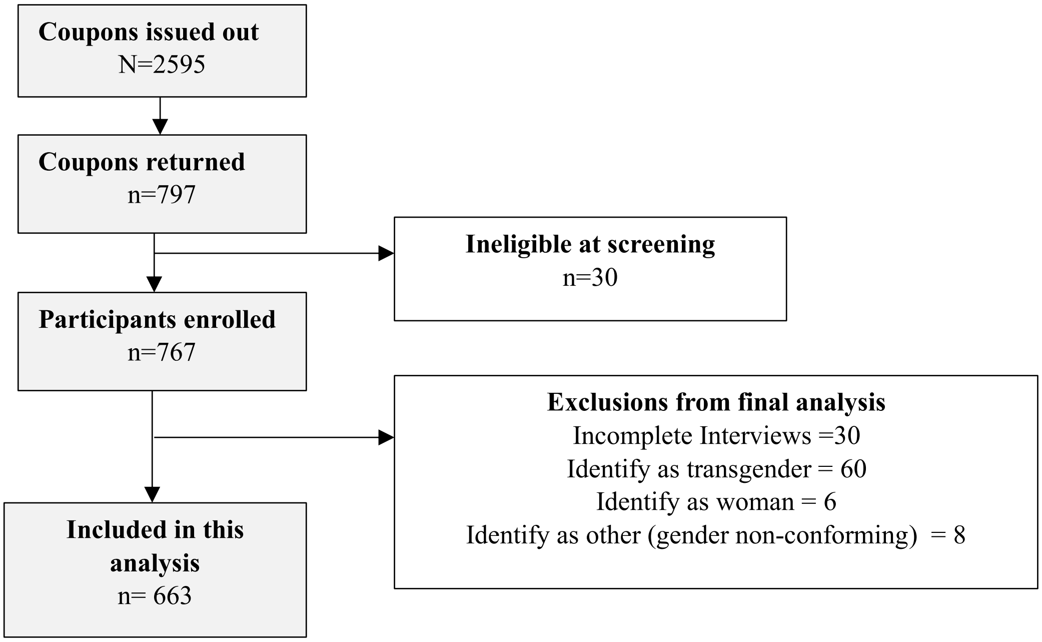

Figure 1: Cape Town recruitment flow diagram

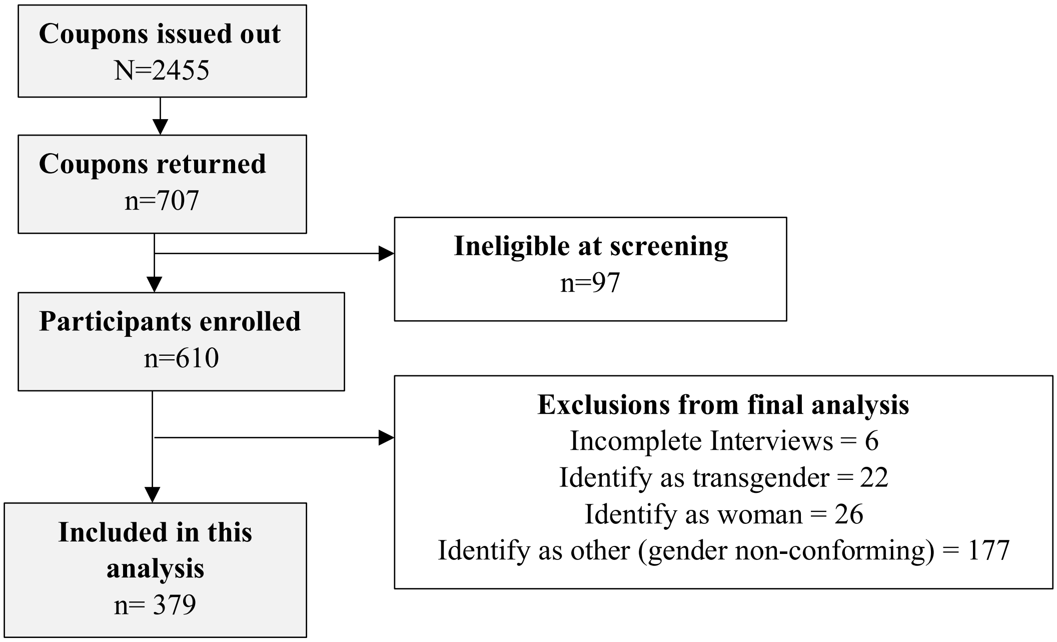

Figure 2: Johannesburg recruitment flow diagram

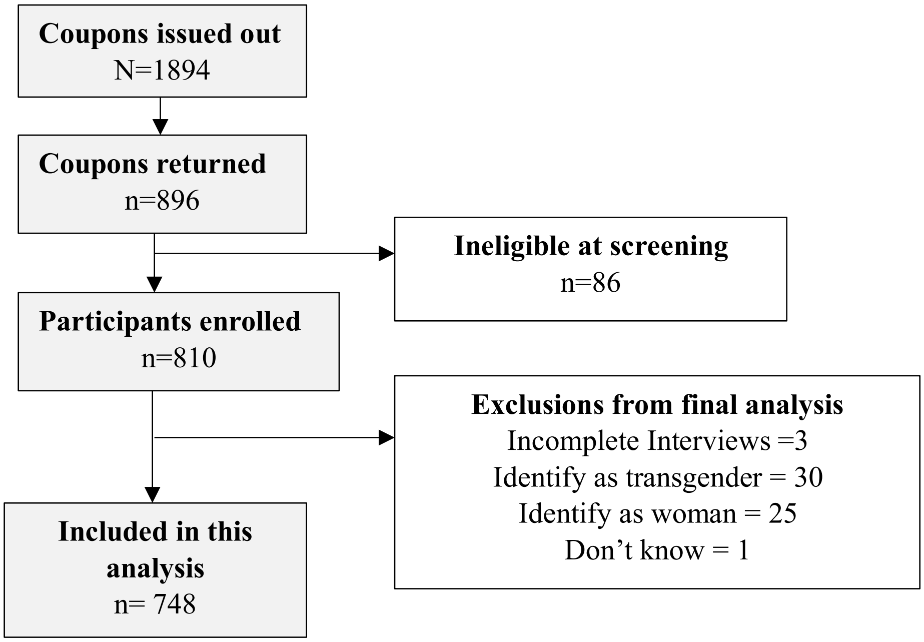

Figure 3: Mahikeng recruitment flow diagram

RDS recruitment trees

Recruitment-driven sampling (RDS) is a method of chain-referral sampling that aims to reach individuals within a specific subpopulation through their social connections. This approach takes into account the size of people's social networks and recruitment patterns to create accurate estimates of population proportions. To mitigate biases arising from variations in network size, statistical weights are assigned based on the sizes of participants' social circles. This approach has been widely used to survey populations that are hard to reach using other sampling techniques. In Figure 4-6 below, we present the recruitment trees from the 6 seeds in Cape Town, 10 seeds in Johannesburg, and 13 seeds in Mahikeng.

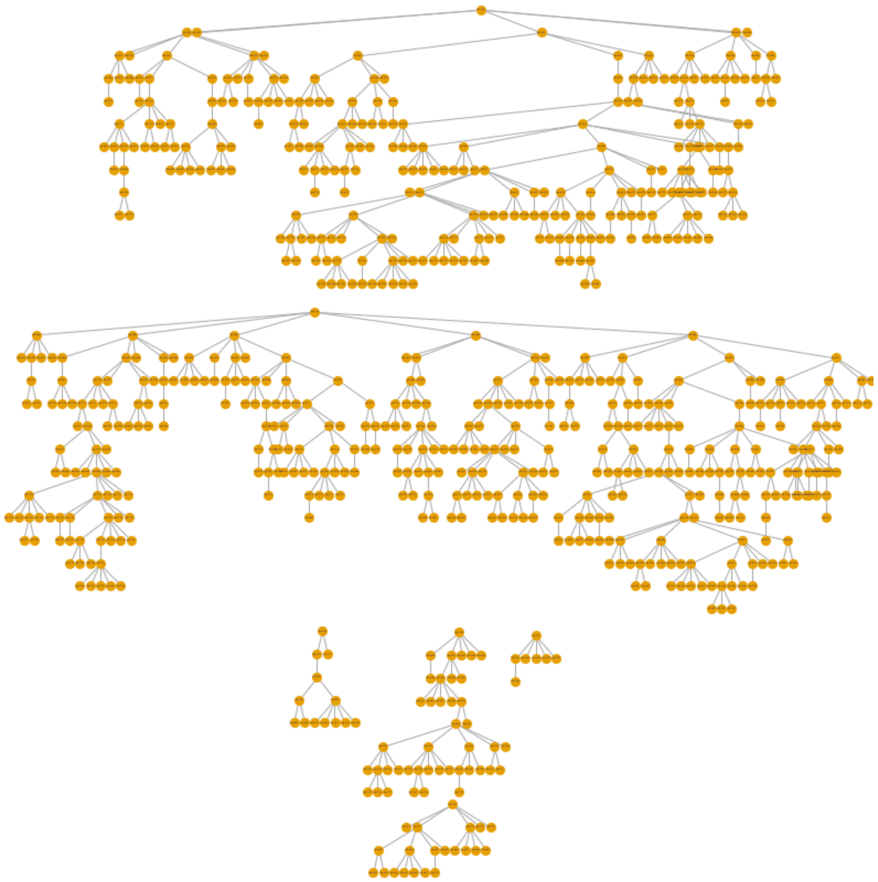

Figure 4: RDS recruitment graph for Cape Town

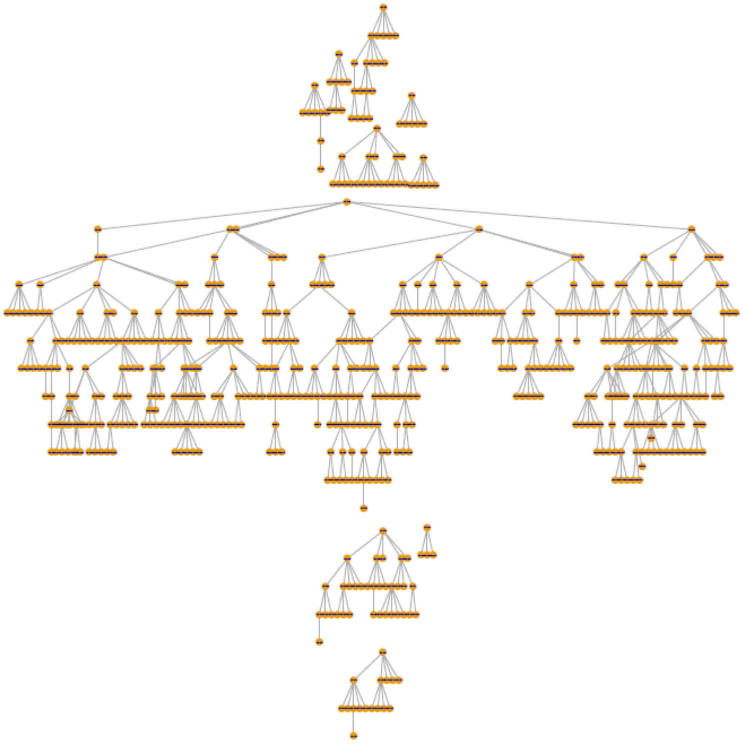

Figure 5: RDS recruitment graph for Johannesburg

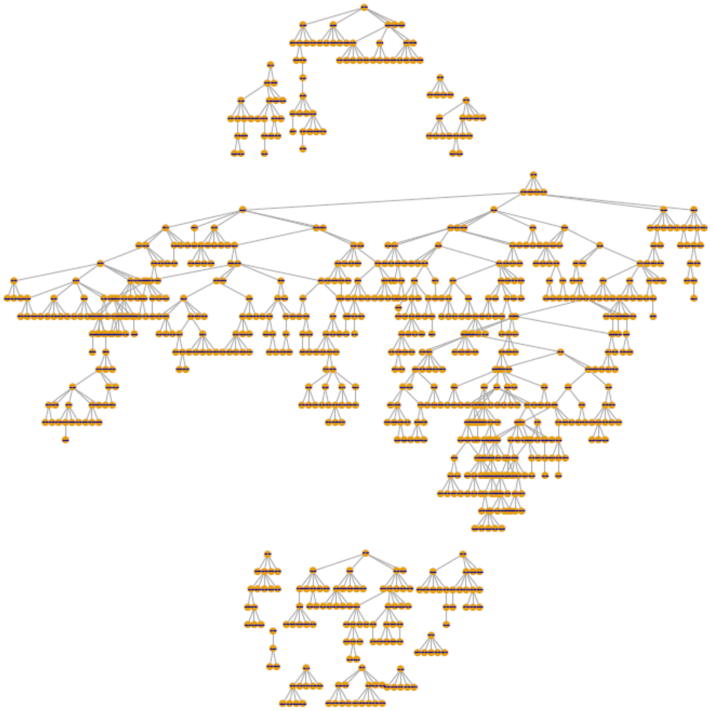

Figure 6: RDS recruitment graph for Johannesburg

Recruitment diagnostics

Figures 2, 3, and 4 illustrate the convergence and bottleneck plots pertaining to HIV prevalence. Bottleneck plots assess the mixing of recruitment trees within the population. Each line represents a prevalence estimate derived from observations originating from a single seed, utilizing only the initial set of observations (as determined by the x-axis). Initial noise in the lines is common due to the limited number of observations contributing to the estimates, but divergence over time may suggest that the trees are traversing distinct, disconnected sub-populations. Convergence plots demonstrate the estimated value using the first number of recruited subjects (defined by the x-axis). Similar to bottleneck plots, initial noise may be observed, but the line should stabilize around the true value as recruitment progresses.

In this survey, the bottleneck plots exhibit no anomalous behavior, with lines generally converging as sample sizes increase (especially for n > 30). Convergence plots for Cape Town and Mahikeng exhibit favourable convergence to the final estimate. In contrast, Johannesburg displays a moderate downward trend throughout recruitment, indicating potentially weaker convergence.

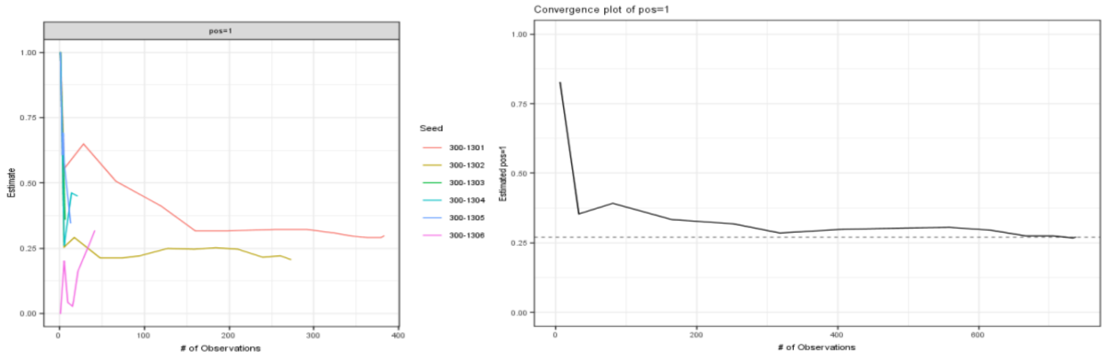

Figure 7: Bottleneck and convergence plots of HIV prevalence in Cape Town

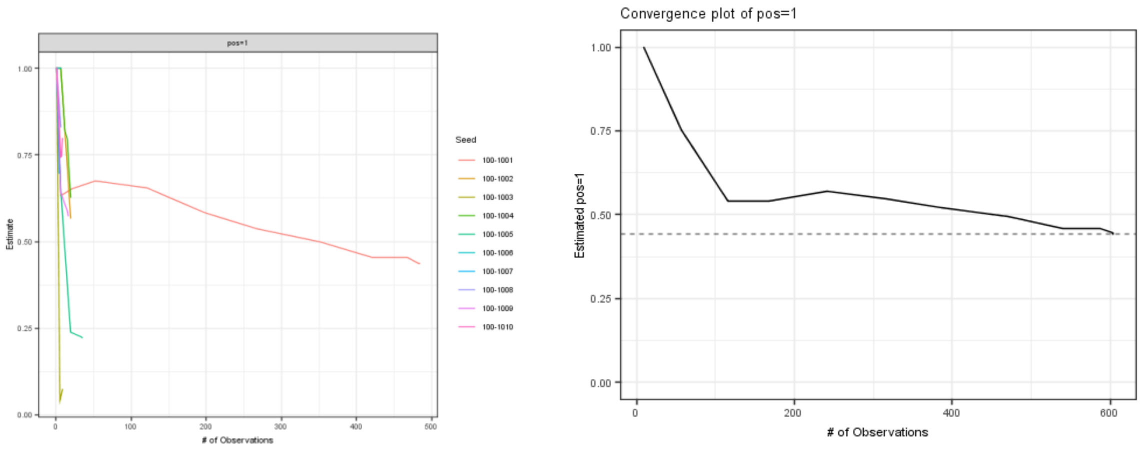

Figure 8: Bottleneck and convergence plots of HIV prevalence in Johannesburg

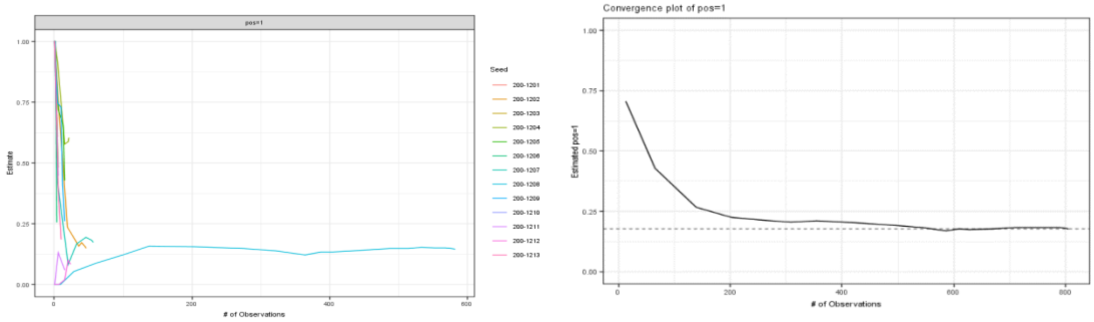

Figure 9: Bottleneck and convergence plots of HIV prevalence in Mahikeng

Convergence plots for HIV cascade by city

The convergence plots for the cascade of care by survey city are displayed in Figures 8, 9, and 10. Each plot exhibits good convergence with the estimation remaining near or oscillating around the final estimate. However, there are possible exceptions in the third 95 in Cape town and the first 95 in Johannesburg where the estimates may show some upward trend until the end of the study. Due to smaller sample sizes associated with the cascade steps, convergence plots may show more variability. Constructing bottleneck plots by breaking down the plots by seed leads to line plots with sample sizes that are too low to be meaningful. Hence, they are not reported here.

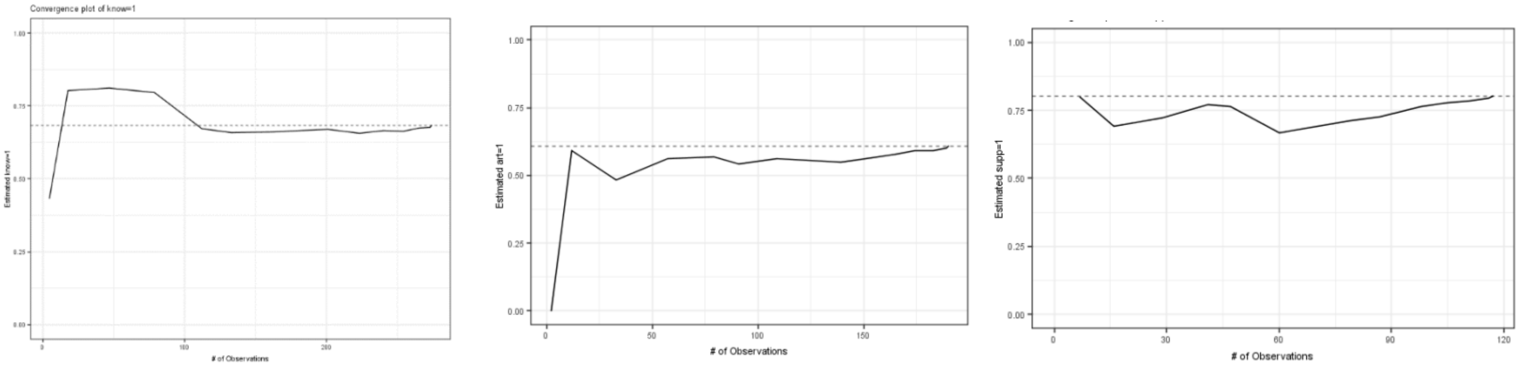

First 95 **Second 95** **Third 95**

Figure 10: Convergence plot of the 95-95-95 care cascade for Cape Town

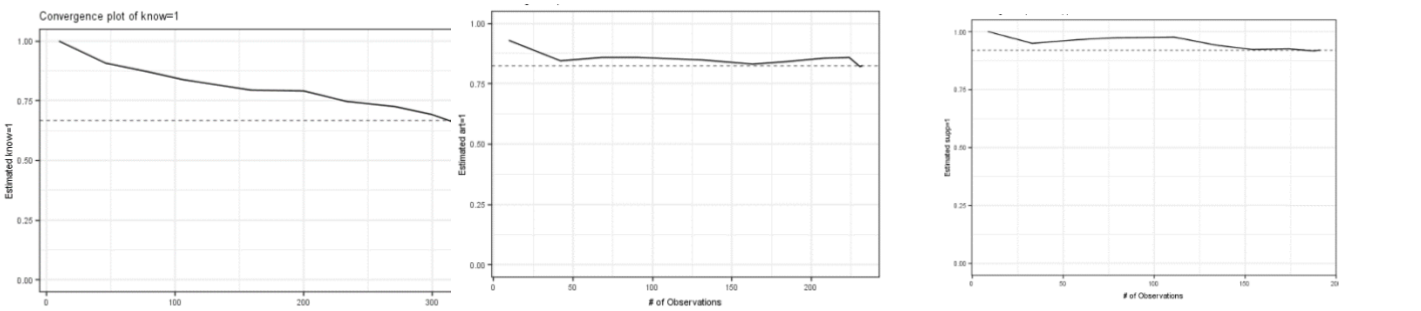

First 95 **Second 95** **Third 95**

Figure 11: Convergence plot of the 95-95-95 care cascade for Johannesburg

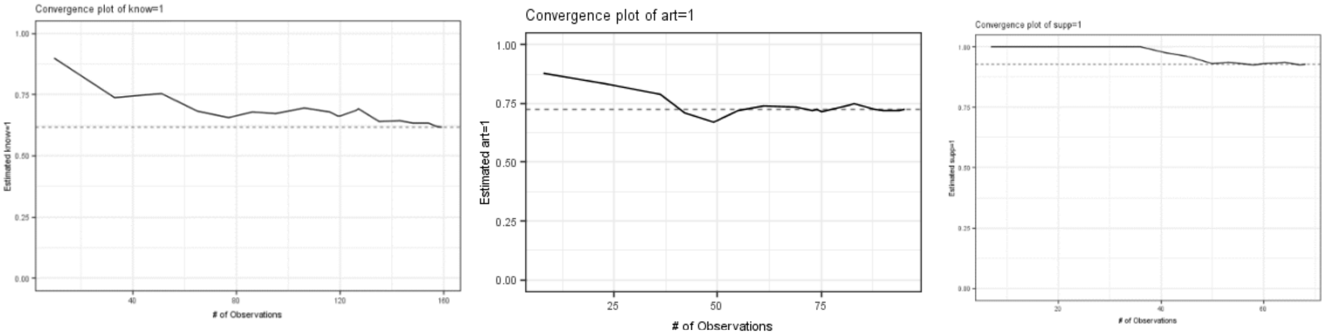

First 95 **Second 95** **Third 95**

Figure 12: Convergence plot of the 95-95-95 care cascade for Johannesburg

Supplementary tables

Table 1: Cascade Variables by Participant Characteristics - Cape Town (Unweighted)

	HIV Prevalence	1st 95	2nd 95	3rd 95
**Characteristic**	**HIV positive**, N = 226[1]	**Aware of HIV positive status**, N = 157[1]	**Aware of status & on ART**, N = 104[1]	**On ART & virally suppressed**, N = 92[1]
Age group				
18-24	51 / 197 (25·9%)	32 / 51 (62·7%)	23 / 32 (71·9%)	20 / 23 (87·0%)
25-34	96 / 255 (37·6%)	67 / 96 (69·8%)	50 / 66 (75·8%)	47 / 50 (94·0%)
35+	79 / 211 (37·4%)	58 / 79 (73·4%)	31 / 56 (55·4%)	25 / 31 (80·6%)
Race				
Black	170 / 397 (42·8%)	118 / 170 (69·4%)	88 / 117 (75·2%)	78 / 88 (88·6%)
Coloured	44 / 216 (20·4%)	28 / 44 (63·6%)	9 / 26 (34·6%)	8 / 9 (88·9%)
Other	12 / 50 (24·0%)	11 / 12 (91·7%)	7 / 11 (63·6%)	6 / 7 (85·7%)
Country of birth				
South Africa	200 / 586 (34·1%)	141 / 200 (70·5%)	92 / 138 (66·7%)	80 / 92 (87·0%)
Other	26 / 77 (33·8%)	16 / 26 (61·5%)	12 / 16 (75·0%)	12 / 12 (100·0%)
Highest level of education completed				
Less than secondary education	95 / 307 (30·9%)	65 / 95 (68·4%)	40 / 63 (63·5%)	31 / 40 (77·5%)
Secondary education	93 / 272 (34·2%)	65 / 93 (69·9%)	42 / 64 (65·6%)	39 / 42 (92·9%)
Tertiary education	38 / 82 (46·3%)	27 / 38 (71·1%)	22 / 27 (81·5%)	22 / 22 (100·0%)
Income in past month				
No monthly income	168 / 498 (33·7%)	114 / 168 (67·9%)	71 / 112 (63·4%)	63 / 71 (88·7%)
Any monthly income	58 / 165 (35·2%)	43 / 58 (74·1%)	33 / 42 (78·6%)	29 / 33 (87·9%)

[1] n / N (%)

Key: 1st 95 = the estimated proportion of participants living with HIV who were aware of their HIV-positive status; 2nd 95 = the estimated proportion of participants who know their status and are on ART; and 3rd 95 = the estimated proportion of participants who are on ART and virally suppressed.

Table 2: Cascade Variables by Participant Characteristics - Cape Town (Weighted)

	HIV Prevalence	1st 95	2nd 95	3rd 95
**Characteristic**	**HIV positive**, N = 7,070[1,2]	**Aware of HIV positive status**, N = 4,735[1,2]	**Aware of status & on ART**, N = 3,051[1,2]	**On ART & virally suppressed**, N = 2,421[1,2]
Age group				
18-24	18·3% (12·6%, 26·0%)	53·9% (35·9%, 71·0%)	58·1% (35·4%, 77·8%)	70·3% (36·0%, 90·9%)
25-34	27·6% (20·5%, 36·0%)	68·2% (50·9%, 81·7%)	71·2% (51·8%, 85·0%)	96·3% (87·8%, 98·9%)
35+	28·6% (20·9%, 37·9%)	73·2% (55·9%, 85·4%)	61·5% (41·5%, 78·2%)	61·8% (28·4%, 86·8%)
Race				
Black	34·9% (28·5%, 41·9%)	66·5% (54·5%, 76·6%)	68·4% (54·8%, 79·4%)	86·2% (73·0%, 93·6%)
Coloured	13·1% (8·0%, 20·7%)	65·4% (42·3%, 82·9%)	50·2% (19·7%, 80·6%)	36·7% (6·4%, 82·9%)
Other	10·7% (5·2%, 20·8%)	86·8% (45·0%, 98·2%)	65·2% (29·7%, 89·3%)	81·0% (32·4%, 97·4%)
Country of birth				
South Africa	23·9% (19·5%, 28·8%)	70·1% (60·3%, 78·4%)	63·6% (50·8%, 74·7%)	75·7% (53·8%, 89·3%)
Other	35·1% (20·7%, 52·9%)	51·5% (24·4%, 77·7%)	74·9% (44·0%, 91·9%)	100·0% (100·0%, 100·0%)
Highest level of education completed				
Less than secondary education	22·6% (16·9%, 29·5%)	66·9% (50·8%, 79·7%)	60·4% (42·1%, 76·2%)	61·1% (33·7%, 82·8%)
Secondary education	27·9% (21·2%, 35·8%)	69·2% (53·9%, 81·1%)	69·1% (52·5%, 81·9%)	95·2% (83·6%, 98·7%)
Tertiary education	33·1% (20·2%, 49·1%)	60·3% (35·4%, 80·8%)	73·7% (43·0%, 91·3%)	100·0% (100·0%, 100·0%)
Income in past month				
No monthly income	25·6% (20·8%, 31·2%)	68·4% (57·6%, 77·5%)	61·6% (47·8%, 73·7%)	76·6% (51·8%, 90·9%)
Any monthly income	23·8% (15·5%, 34·8%)	61·6% (36·1%, 82·0%)	79·7% (60·7%, 90·9%)	88·1% (69·1%, 96·1%)

[1] % (95% CI)

[2] CI = Confidence Interval

Key: 1st 95 = the estimated proportion of MSM living with HIV who were aware of their HIV-positive status; 2nd 95 = the estimated proportion of MSM who know their status and are on ART; and 3rd 95 = the estimated proportion of MSM who are on ART and virally suppressed.

Table 3: Cascade Variables by Participant Characteristics – Johannesburg (Unweighted)

Characteristic	HIV Prevalence<br>HIV positive, N = 181[1]	1st 95<br>Aware of HIV positive status, N = 135[1]	2nd 95<br>Aware of status & on ART, N = 105[1]	3rd 95<br>On ART & virally suppressed, N = 94[1]
Age group				
18-24	36 / 123 (29·3%)	26 / 36 (72·2%)	21 / 25 (84·0%)	19 / 21 (90·5%)
25-34	88 / 163 (54·0%)	62 / 88 (70·5%)	45 / 62 (72·6%)	39 / 45 (86·7%)
35+	57 / 93 (61·3%)	47 / 57 (82·5%)	39 / 47 (83·0%)	36 / 39 (92·3%)
Race				
Black	176 / 362 (48·6%)	132 / 176 (75·0%)	104 / 131 (79·4%)	93 / 104 (89·4%)
Coloured	4 / 12 (33·3%)	2 / 4 (50·0%)	1 / 2 (50·0%)	1 / 1 (100·0%)
Other	1 / 5 (20·0%)	1 / 1 (100·0%)	0 / 1 (0·0%)	
Country of birth				
South Africa	155 / 326 (47·5%)	113 / 155 (72·9%)	90 / 112 (80·4%)	80 / 90 (88·9%)
Other	26 / 53 (49·1%)	22 / 26 (84·6%)	15 / 22 (68·2%)	14 / 15 (93·3%)
Highest level of education completed				
Less than secondary education	38 / 70 (54·3%)	30 / 38 (78·9%)	26 / 30 (86·7%)	22 / 26 (84·6%)
Secondary education	122 / 264 (46·2%)	90 / 122 (73·8%)	66 / 89 (74·2%)	61 / 66 (92·4%)
Tertiary education	21 / 45 (46·7%)	15 / 21 (71·4%)	13 / 15 (86·7%)	11 / 13 (84·6%)
Income in past month				
Any monthly income	104 / 198 (52·5%)	82 / 104 (78·8%)	67 / 81 (82·7%)	63 / 67 (94·0%)
No monthly income	77 / 181 (42·5%)	53 / 77 (68·8%)	38 / 53 (71·7%)	31 / 38 (81·6%)

[1] n / N (%)

Key: 1st 95 = the estimated proportion of participants living with HIV who were aware of their HIV-positive status; 2nd 95 = the estimated proportion of participants who know their status and are on ART; and 3rd 95 = the estimated proportion of participants who are on ART and virally suppressed.

Table 4: Cascade Variables by Participant Characteristics – Johannesburg (Weighted)

Characteristic	HIV Prevalence<br>HIV positive, N = 9,841[1,2]	1st 95<br>Aware of HIV positive status, N = 6,668[1,2]	2nd 95<br>Aware of status & on ART, N = 5,171[1,2]	3rd 95<br>On ART & virally suppressed, N = 4,585[1,2]
Age group				
18-24	24·1% (15·5%, 35·5%)	63·4% (35·8%, 84·3%)	86·5% (66·2%, 95·5%)	85·4% (53·7%, 96·7%)
25-34	43·6% (34·0%, 53·7%)	71·3% (57·1%, 82·2%)	71·6% (54·1%, 84·3%)	86·6% (64·9%, 95·7%)
35+	55·2% (42·0%, 67·8%)	66·0% (46·1%, 81·5%)	81·5% (62·0%, 92·3%)	92·5% (76·0%, 98·0%)
Race				
Black	41·1% (34·7%, 47·7%)	69·9% (59·1%, 78·9%)	78·4% (67·4%, 86·4%)	88·6% (77·5%, 94·6%)
Coloured	47·4% (13·5%, 83·9%)	12·2% (1·4%, 58·4%)	65·1% (10·2%, 96·9%)	100·0% (100·0%, 100·0%)
Other	7·2% (0·6%, 48·5%)	100·0% (100·0%, 100·0%)	0·0% (0·0%, 0·0%)	
Country of birth				
South Africa	39·8% (33·0%, 47·1%)	64·1% (51·8%, 74·8%)	81·6% (70·6%, 89·1%)	86·0% (72·6%, 93·4%)
Other	44·6% (29·7%, 60·4%)	82·4% (55·2%, 94·6%)	66·7% (39·7%, 85·9%)	98·8% (91·3%, 99·9%)
Highest level of education completed				
Less than secondary education	48·6% (34·7%, 62·7%)	70·4% (47·4%, 86·3%)	79·0% (50·7%, 93·2%)	76·2% (47·4%, 91·9%)
Secondary education	39·8% (32·2%, 47·9%)	65·9% (52·0%, 77·6%)	75·7% (62·8%, 85·2%)	93·0% (80·8%, 97·7%)
Tertiary education	30·2% (17·2%, 47·4%)	75·8% (50·5%, 90·5%)	91·4% (66·4%, 98·3%)	94·3% (77·3%, 98·8%)
Income in past month				
Any monthly income	45·0% (35·9%, 54·5%)	67·1% (51·0%, 79·9%)	79·0% (63·5%, 89·0%)	94·4% (83·0%, 98·3%)
No monthly income	36·0% (27·8%, 45·1%)	68·7% (53·5%, 80·7%)	76·5% (60·0%, 87·6%)	80·9% (59·7%, 92·4%)

[1] % (95% CI)

[2] CI = Confidence Interval

Key: 1st 95 = the estimated proportion of MSM living with HIV who were aware of their HIV-positive status; 2nd 95 = the estimated proportion of MSM who know their status and are on ART; and 3rd 95 = the estimated proportion of MSM who are on ART and virally suppressed.

Table 5: Cascade Variables by Participant Characteristics - Mahikeng (Unweighted)

	HIV Prevalence	1st 95	2nd 95	3rd 95
**Characteristic**	**HIV positive**, N = 125[1]	**Aware of HIV positive status**, N = 74[1]	**Aware of status & on ART**, N = 53[1]	**On ART & virally suppressed**, N = 48[1]
Age group				
18-24	26 / 342 (7·6%)	13 / 26 (50·0%)	9 / 13 (69·2%)	8 / 9 (88·9%)
25-34	62 / 295 (21·0%)	36 / 62 (58·1%)	23 / 35 (65·7%)	21 / 23 (91·3%)
35+	37 / 111 (33·3%)	25 / 37 (67·6%)	21 / 25 (84·0%)	19 / 21 (90·5%)
Race				
Black	117 / 712 (16·4%)	68 / 117 (58·1%)	48 / 67 (71·6%)	43 / 48 (89·6%)
Coloured	7 / 35 (20·0%)	6 / 7 (85·7%)	5 / 6 (83·3%)	5 / 5 (100·0%)
Other	1 / 1 (100·0%)	0 / 1 (0·0%)		
Country of birth				
South Africa	122 / 738 (16·5%)	72 / 122 (59·0%)	51 / 71 (71·8%)	46 / 51 (90·2%)
Other	3 / 10 (30·0%)	2 / 3 (66·7%)	2 / 2 (100·0%)	2 / 2 (100·0%)
Highest level of education completed				
Less than secondary education	28 / 139 (20·1%)	17 / 28 (60·7%)	13 / 17 (76·5%)	11 / 13 (84·6%)
Secondary education	73 / 455 (16·0%)	37 / 73 (50·7%)	27 / 36 (75·0%)	25 / 27 (92·6%)
Tertiary education	24 / 152 (15·8%)	20 / 24 (83·3%)	13 / 20 (65·0%)	12 / 13 (92·3%)
Income in past month				
No monthly income	65 / 510 (12·7%)	39 / 65 (60·0%)	28 / 38 (73·7%)	27 / 28 (96·4%)
Any monthly income	60 / 238 (25·2%)	35 / 60 (58·3%)	25 / 35 (71·4%)	21 / 25 (84·0%)

[1] n / N (%)

Key: 1st 95 = the estimated proportion of participants living with HIV who were aware of their HIV-positive status; 2nd 95 = the estimated proportion of participants who know their status and are on ART; and 3rd 95 = the estimated proportion of participants who are on ART and virally suppressed.

Table 6: Cascade Variables by Participant Characteristics - Mahikeng (Weighted)

	HIV Prevalence	1st 95	2nd 95	3rd 95
**Characteristic**	**HIV positive**, N = 524[1,2]	**Aware of HIV positive status**, N = 316[1,2]	**Aware of status & on ART**, N = 219[1,2]	**On ART & virally suppressed**, N = 199[1,2]
Age group				
18-24	5·5% (3·6%, 8·2%)	48·5% (28·9%, 68·6%)	73·7% (44·5%, 90·7%)	78·1% (29·3%, 96·8%)
25-34	17·7% (13·4%, 23·0%)	56·6% (42·0%, 70·0%)	58·2% (38·7%, 75·5%)	89·0% (63·5%, 97·4%)
35+	31·6% (22·2%, 42·8%)	69·9% (50·3%, 84·1%)	84·6% (57·1%, 95·8%)	95·1% (80·8%, 98·9%)
Race				
Black	14·8% (12·0%, 18·0%)	59·7% (48·9%, 69·6%)	71·8% (57·1%, 83·0%)	90·2% (76·5%, 96·3%)
Coloured	11·7% (5·2%, 24·2%)	82·1% (34·9%, 97·5%)	84·9% (38·4%, 98·1%)	100·0% (100·0%, 100·0%)
Other	100·0% (100·0%, 100·0%)	0·0% (0·0%, 0·0%)		
Country of birth				
South Africa	14·5% (11·9%, 17·7%)	60·7% (50·1%, 70·4%)	72·0% (57·7%, 82·9%)	90·5% (77·3%, 96·4%)
Other	26·5% (6·6%, 64·7%)	39·7% (5·4%, 88·3%)	100·0% (100·0%, 100·0%)	100·0% (100·0%, 100·0%)
Highest level of education completed				
Less than secondary education	20·7% (13·6%, 30·1%)	59·0% (36·7%, 78·2%)	87·8% (67·8%, 96·1%)	92·4% (71·1%, 98·4%)
Secondary education	12·5% (9·6%, 16·1%)	53·8% (40·2%, 66·8%)	69·8% (47·4%, 85·6%)	91·8% (70·8%, 98·1%)
Tertiary education	15·9% (10·3%, 23·7%)	80·3% (55·5%, 93·0%)	60·2% (34·5%, 81·3%)	85·6% (41·9%, 98·0%)
Income in past month				
No monthly income	11·0% (8·3%, 14·4%)	60·8% (46·4%, 73·5%)	76·0% (57·3%, 88·1%)	97·2% (81·7%, 99·6%)
Any monthly income	22·2% (16·8%, 28·9%)	59·7% (44·4%, 73·3%)	69·2% (47·6%, 84·7%)	83·9% (61·0%, 94·6%)

[1] % (95% CI)

[2] CI = Confidence Interval

Key: 1st 95 = the estimated proportion of MSM living with HIV who were aware of their HIV-positive status; 2nd 95 = the estimated proportion of MSM who know their status and are on ART; and 3rd 95 = the estimated proportion of MSM who are on ART and virally suppressed.
