# Supplementary material for: The treatment gap and the HIV care continuum for cisgender men who have sex with men in three South African cities: Findings from a biobehavioural survey, 2019
Source: PLOS Glob Public Health. 2025 Sep 2;5(9):e0004874. doi: 10.1371/journal.pgph.0004874 (PMC12404541; doi:10.1371/journal.pgph.0004874)
Supplement: Attachment — Submitted filename: Response to reviewers.pdf [file pgph.0004874.s006.pdf]

Manuscript PGPH-D-24-02814 – Response to Reviewers

Dear Editorial and Review Team,

We appreciate the thoughtful comments of the reviewers. Responses to the comments are provided below and revisions have been incorporated into the revised manuscript. Please let us know if we can provide any additional clarifications or edits.

REVIEWER #1: The manuscript is well written, and it reads well. There are no discrepancies in the statistical analysis. The results are well presented, and I do believe this manuscript meets PLOS one standards. Thank you.

REVIEWER #2: Thank you for the opportunity to review this important and timely manuscript. Overall, the manuscript is well written. The methods are well described and easy to follow. The comments below are offered in the spirit of collegiality to strengthen the manuscript.

Introduction

- To frame SA's HIV epidemic please provide the case rate rather than incidence so that readers can contextualize within the global HIV epidemic.

<u>Response</u>: We have edited the first sentence: *"South Africa has the largest proportion of people living with HIV globally with approximately 7.6 million people living with HIV in 2022 (among 39.5 million people estimated to be living with HIV globally) (1) and an estimated HIV prevalence of 12.7% across all age groups and 17% among adults aged 15-49 years (2)."*

- It seems a bit misleading in the second sentence to frame 95%-95%-95% as a country specific HIV program goal rather than a global UNAID initiative particularly when neighboring countries have nearly achieved these goals.

<u>Response</u>: We have edited this sentence for make it clear this is a global target.

- In line 29, please specify where these studies are done, in SA? Globally? Also which HIV outcomes are the authors referencing?

<u>Response</u>: We have decided to remove this sentence and expand on the following sentences where we go into more detail on specific studies.

- Line 30 - the authors have not defined SSA

<u>Response</u>: Corrected, thank you!

Methods

- Why was undetectable defined as vl<1000? At the time the study was conducted, I believe the South African ART guidelines defined undetectable as <400. This requires an explanation.

<u>Response</u>: It is correct that the South African National Department of Health defined viral suppression at a lower threshold (i.e., a viral load of ≤50 copies/mL). In addition, virologic failure was defined as two consecutive viral load results ≥1000 copies/mL. In our study, we used the <1000 copies/mL threshold to denote viral suppression for two main reasons. First, this threshold aligns with the programmatic definition of virologic failure used in routine HIV care in South Africa and thus reflects clinical decision-making in real-world settings. Second, we aimed to ensure comparability with other South African bio-behavioural surveys that applied the same threshold to define viral suppression, enabling more meaningful comparisons across findings. We have added a sentence explaining this to the Measure section of the Methods.

Results

- Given that 707 issued coupons in Joburg were returned and none declined to participate, how were so many excluded from Joburg to include a sample of only 379? Were most /all TGW and gender non-conforming individuals there? If so, please state.

Response: In Figure 2 of Appendix 2 (Supplementary Material), we provide a flow chart of the exclusions from the 707 individuals who returned the coupons. We note that 97 were ineligible at screening. For this analysis, of the 619 enrolled: 6 had incomplete interviews, 22 identified as transgender, 26 identified as women, and 177 as gender non-conforming.

Discussion

- The third paragraph of the discussion needs more clarity. Reviewing the related study about factors associated with VS among MSM is prudent, but the authors go on to discuss the social ecological model (which is not an intersectional model) then go on to very casually toss in discussion about intersectional stigma and its multifaceted manifestations, syndemic theory, and structural discrimination. These are all huge topics that should not be unwrapped for a single toss away sentence. The authors should either contend with these theories and constructs appropriately or stick to the social ecological model alone.

Response: we have revised the third paragraph of the discussion to more clearly focus on the social-ecological framework. We removed the brief references to intersectional stigma, syndemic theory, and structural discrimination, as these are indeed complex constructs that warrant more in-depth treatment than a single sentence allows. Instead, we now focus on the multilevel factors that shape HIV outcomes for MSM, including individual behaviors, social relationships, community norms, and structural barriers. We also clarified the relevance of structural interventions for addressing gaps in the HIV care continuum, ensuring the paragraph more clearly supports the main points of our analysis.

- Line 308 the authors bring up an important consideration about dolutegravir. To my recollection, SA began using dolutegravir as a second line agent for suspected treatment failure in October/November 2019 and may be more influential than authors suggest. Did the survey ask any questions about ART regimens or regimen switches that may help to assure that this is not a more significant limitation?

Response: Thank you for your comment. The study did not gather any information about the ART regimens. The reviewer's estimates regarding the duration of treatment (DTG) for second-line antiretroviral therapy (ART) are close. Guidelines were published in October 2019 (the month, the study concluded), but early implementation of guidelines began in December 2019. Therefore, we suggest that without collecting information about ART regimens, it still is a less significant limitation. https://www.health.gov.za/wp-content/uploads/2020/11/2019-art-guideline.pdf

Thank you again for your consideration and review.
